# *Sonic hedgehog* is Essential for Proximal-Distal Outgrowth of the Limb Bud in Salamanders

**DOI:** 10.3389/fcell.2022.797352

**Published:** 2022-04-01

**Authors:** Sruthi Purushothaman, Brianda B. Lopez Aviña, Ashley W. Seifert

**Affiliations:** Department of Biology, University of Kentucky, Lexington, KY, United States

**Keywords:** limb development, salamander, Sonic hedgehog, zone of polarizing activity (ZPA), BMS-833923, CRISPR-Cas9, cell proliferation, cell death

## Abstract

The developing forelimb has been a foundational model to understand how specified progenitor cells integrate genetic information to produce the tetrapod limb bauplan. Although the reigning hypothesis is that all tetrapods develop limbs in a similar manner, recent work suggests that urodeles have evolved a derived mode of limb dvelopment. Here, we demonstrate through pharmacological and genetic inactivation of *Sonic hedgehog* (*Shh*) signaling in axolotls that *Shh* directs expansion and survival of limb progenitor cells in addition to patterning the limb across the proximodistal and antero-posterior axis. In contrast to inactivation of *Shh* in mouse or chick embryos where a humerus, radius, and single digit develop, *Shh* crispant axolotls completely lack forelimbs. In rescuing limb development by implanting SHH-N protein beads into the nascent limb field of *Shh* crispants, we show that the limb field is specified in the absence of *Shh* and that hedgehog pathway activation is required to initiate proximodistal outgrowth. When our results are examined alongside other derived aspects of salamander limb development and placed in a phylogenetic context, a new hypothesis emerges whereby the ability for cells at an amputation plane to activate morphogenesis and regenerate a limb may have evolved uniquely in urodeles.

## Introduction

Genetic and molecular investigation of early amniote forelimb and pectoral fin development has revealed a high degree of mechanistic conservation across the relatively few model organisms that have been well studied (e.g., mouse, chick, *Xenopus*, and zebrafish). Forelimb development in amniote embryos, and, to a large extent, pectoral fin development, can be deconstructed into four general phases: progenitor field establishment and positioning; initiation and expansion of limb progenitor cells; patterning of limb progenitors across three cardinal axes (anteroposterior, proximodistal, and dorsoventral); and morphogenesis of the mesoderm into a mature limb integrating muscles, skeletal elements, and connective tissues (Mercader, 2007; Zeller et al., 2009; McQueen and Towers, 2020). Forelimb and pectoral fin field establishment begins when retinoic acid (RA) specifies a subpopulation of the somatopleure (Helms et al., 1996; Tulenko et al., 2013; Gros and Tabin, 2014; Nishimoto et al., 2015) to become forelimb/fin mesoderm, whereas *Hox* gene expression aligns these progenitors along the craniocaudal (head-to-tail) axis (Rancourt et al., 1995; Moreau et al., 2019). Forelimb and pectoral fin bud initiation occurs when RA and canonical Wnt signaling subsequently induce *Tbx5* among forelimb/pectoral fin field progenitors (Ahn et al., 2002; Garrity et al., 2002; Grandel et al., 2002; Agarwal et al., 2003; Gibert et al., 2006; Nishimoto et al., 2015). Limb and fin bud outgrowth occurs when *Fgf10* is activated throughout the nascent bud mesoderm, which induces fibroblast growth factor (Fgf) signaling in the overlying ectoderm to create positive feedback between the ectoderm and mesoderm (Xu et al., 1998; Ohuchi et al., 2000; Kawakami et al., 2004; Norton et al., 2005; Yu and Ornitz, 2008). As the forelimb and pectoral fin bud emerge from the body wall, it acquires anteroposterior polarity with *Hand2*, and several *HoxA/D* genes restricted to the posterior and *Gli3* expression portioned into the anterior mesoderm (te Welscher et al., 2002; Charité et al., 2000; Kmita et al., 2005; Tarchini et al., 2006; Sordino et al., 1995). Subsequently, two signaling centers form that control limb development along the proximodistal and anteroposterior axes, respectively: the apical ectodermal ridge (AER), marked primarily by *Fgf8* expression, and the zone of polarizing activity (ZPA), marked by *Sonic hedgehog* (*Shh*) expression (Saunders, 1948; Saunders et al., 1962; Echelard et al., 1993; Riddle et al., 1993; Heikinheimo et al., 1994; Crossley et al., 1996a).

AER excisions in chicken embryos and gene knockout experiments in mice demonstrated that Fgfs secreted from the AER are essential for limb development where they promote cell survival and proximodistal outgrowth of the limb (Saunders, 1948; Lewandoski et al., 2000; Sun et al., 2000; Mariani et al., 2008). Inactivation of *Fgfs 4*, *8*, and *9* in the ectoderm (Mariani et al., 2008) or early removal of limb bud ectoderm results in a scapula alone (Saunders, 1948). Similarly, the role of the ZPA and Shh signaling has been extensively studied during chicken and mouse limb development. Following outgrowth and establishment of the AER, Shh sets up anteroposterior positional values (Riddle et al., 1993; Yang et al., 1997; Zhu et al., 2008), maintains AER width and expression of the AER-*Fgfs* (*via Gremlin1* restriction of Bmp signaling) (Laufer et al., 1994; Niswander et al., 1994; Kraus et al., 2001; Ros et al., 2003; Harfe et al., 2004; Scherz et al., 2007), and regulates cell proliferation (Cooke and Summerbell, 1980; Towers et al., 2008; Towers et al., 2011) and cell survival of limb mesoderm (Sanz-Ezquerro and Tickle, 2000). The spatial restriction of AER-*Fgfs* and *Shh* has been analyzed in a spectrum of vertebrate species supporting conserved expression in the ectoderm and mesoderm respectively (Echelard et al., 1993; Riddle et al., 1993; Heikinheimo et al., 1994; Crossley et al., 1996a; Christen and Slack, 1998; Neumann et al., 1999; Leal and Cohn, 2016).

Despite this apparent conservation, previous work has shown that salamanders lack an AER (Sturdee and Connock, 1975; Tank et al., 1977) and that at some point during amphibian evolution localization of *Fgfs* and *Fgf* receptors shifted to the limb mesenchyme where they now control limb size but are largely dispensable for limb development (Purushothaman et al., 2019). At the very least, these data suggest that the molecular logic of tetrapod limb development may not be absolutely conserved among all tetrapods. Here, we asked whether reduced prominence of Fgf signaling during salamander limb development might be offset by an increased reliance on Shh signaling to control limb field progenitor proliferation, survival, outgrowth, and patterning. To test our hypothesis, we pharmacologically inhibited Shh signaling in axolotls using cyclopamine or the highly specific smoothened antagonist BMS-833923. We also genetically inactivated *Shh* using CRISPR/Cas9 and analyzed limb development in *Shh* crispants. We asked whether SHH-N or FGF-8b protein could stimulate limb development outside the limb field as has been observed in chick embryos (Cohn et al., 1995) and whether the limb field formed in the absence of Shh signaling.

## Results

### Small-Molecule Smoothened Antagonist BMS-833923 Inhibits Proximodistal Outgrowth of the Limb Bud in Axolotl Embryos

**FIGURE 1:**
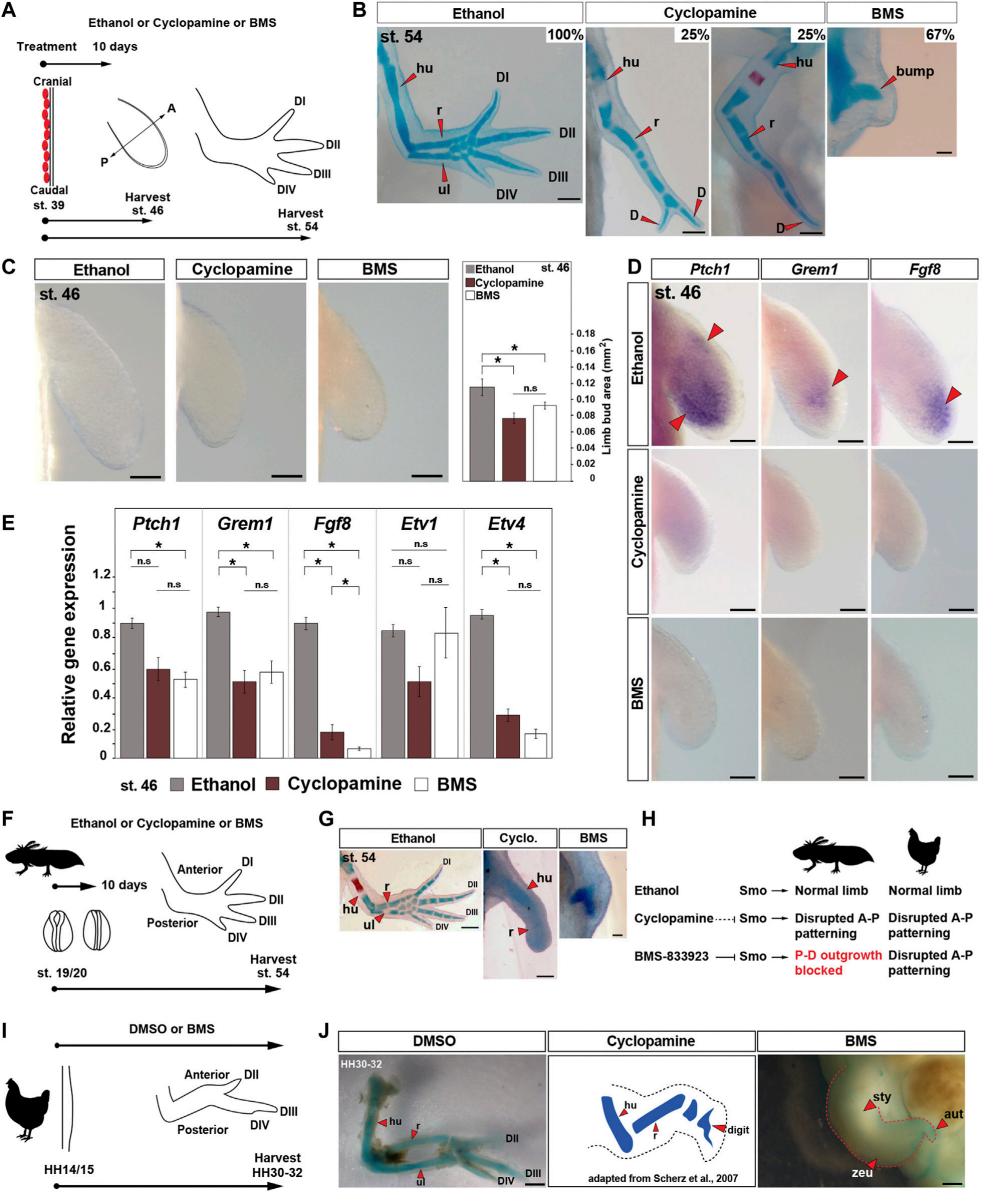
Small-molecule smoothened antagonist BMS-833923 inhibits limb bud outgrowth in axolotl larvae. **(A)** Design for ethanol (control), cyclopamine, and BMS treatments in axolotl. Limbs are aligned with anterior “A” on the top and posterior “P” on the bottom. Red ovals depict dorsal muscle blocks. **(B)** Representative images of Alcian blue/Alizarin red–stained ethanol-, cyclopamine-, or BMS-treated stage 54 limbs (limb *n* = 30 for ethanol, 24 for cyclopamine, and 36 for BMS-833923). Scale bar = 500 µm. **(C)** Limb bud area measurements in ethanol (control)-, cyclopamine-, or BMS-treated limbs [one-way ANOVA, Tukey–Kramer HSD post hoc test, *F* = 8.628; Tukey–Kramer HSD post hoc test, *p* = 0.0026 (ethanol vs. cyclopamine), *p* = 0.046 (ethanol vs. BMS), and *p* = 0.34 (cyclopamine vs. BMS); *n* = 6 per treatment]. Scale bar = 100 µm. **(D)** *In situ* hybridization for genes *Ptch1*, *Grem1*, and *Fgf8* in ethanol-, cyclopamine-, or BMS-treated stage 46 limbs (*n* = 3 or 4 per gene). Red arrows: expression domain. Scale bar = 100 µm. **(E)** qRT-PCR for *Ptch1*, *Grem1*, *Fgf8*, *Etv1*, and *Etv4* expression in stage 46 limbs post ethanol, cyclopamine, or BMS treatments [one-way ANOVA, Tukey–Kramer HSD post hoc test; *Ptch1*: *F* = 7.98, *p* = 0.06 (ethanol vs. cyclopamine), *p* = 0.02 (ethanol vs. BMS), and *p* = 0.65 (cyclopamine vs. BMS); *Grem1*: *F* = 8.65, *p* = 0.018 (ethanol vs. cyclopamine), *p* = 0.048 (ethanol vs. BMS), and *p* = 0.68 (cyclopamine vs. BMS); *Fgf8*: *F* = 301.43, *p* < 0.0001 (ethanol vs. cyclopamine), *p* < 0.0001 (ethanol vs. BMS), and *p* = 0.03 (cyclopamine vs. BMS); *Etv1*: *F* = 2.986, *p* = 0.16 (ethanol vs. cyclopamine), *p* = 0.99 (ethanol vs. BMS), and *p* = 0.17 (cyclopamine vs. BMS); *Etv4*: *F* = 225.92, *p* < 0.0001 (ethanol vs. cyclopamine), *p* < 0.0001 (ethanol vs. BMS), and *p* = 0.13 (cyclopamine vs. BMS); *n* = 3 per treatment]. **(F)** Design for ethanol, cyclopamine, or BMS treatments at neural fold stage 19/20 in axolotls. **(G)** Alcian blue/Alizarin red staining at stage 54 for neural fold treatments with ethanol, cyclopamine, or BMS (*n* = 3 per treatment). Scale bar = 500 µm. **(H)** Schematic depicting the mode of actions of ethanol, cyclopamine, and BMS in axolotl and chick limb buds. **(I)** Design for ethanol, cyclopamine, or BMS treatments at HH14/15 in chick embryos. **(J)** Alcian blue/Alizarin red–stained DMSO, and cyclopamine-treated (adapted from the work of Scherz et al., 2007) and BMS-treated limbs at HH30-32 (*n* = 4 per treatment). Scale bar = 1 mm. Error bars: SEM; and asterisk: significant *p*-values. hu, humerus; r, radius; ul, ulna; D, digit; sty, stylopod; zeu, zeugopod; and aut, autopod.

To test the hypothesis that Shh signaling coordinates expansion of limb field progenitor cells and proximodistal outgrowth of the limb bud in salamanders, we first inhibited Shh signaling throughout early limb development using a pharmacologic approach. Previous studies using cyclopamine during axolotl limb development provided evidence that *Shh* functions primarily to pattern the anteroposterior axis following expansion of limb bud progenitors (Stopper and Wagner, 2007). Curiously, recent work exploring Shh signaling during zebrafish development and caudal fin regeneration revealed that a highly specific smoothened antagonist, BMS-833923 (hereafter BMS) (Akare et al., 2014) more potently and specifically inhibited hedgehog signaling compared to cyclopamine (Armstrong et al., 2017). Therefore, we treated pre-limb bud stage larvae (stage 39) with ethanol, cyclopamine, or BMS for 10 days (initiation and expansion phase) and harvested limbs at stages 46 and 54 (Figures 1A,B and Supplementary Figures S1A–C). Analyzing skeletal differentiation at stage 54, we found cyclopamine treatment primarily affected anteroposterior patterning, with nearly all (∼88%) resultant limbs possessing a humerus, single fused radius/ulna, and at least one digit (Figure 1B and Supplementary Figures S1A, S1B, S1D). These results mirrored previous limb development studies in urodele and amniote embryos using the same concentration of cyclopamine (Omnell et al., 1990; Scherz et al., 2007; Stopper and Wagner, 2007). In stark contrast, 92% of the BMS-treated larvae had no limbs with only a small bump covering the scapula where the humerus would normally articulate or no bump at all (Figure 1B and Supplementary Figures S1C, S1D).

Examining pre-chondrogenic limbs at stage 46, we observed that treatment with cyclopamine or BMS caused a significant decrease in limb bud size, although small limb buds still formed in both treatment groups (Figure 1C). To assess the degree to which these drugs inhibited Shh signaling, we assessed expression of the direct target gene *Patched1* (*Ptch1*) and downstream target genes, *Gremlin1* (*Grem1*) and *Fibroblast growth factor 8* (*Fgf8*) in stage 46 limb buds (Figures 1D,E). Using *in situ* hybridization and qRT-PCR, we observed that *Ptch1* was significantly downregulated by BMS treatment, *Grem1* was significantly downregulated in BMS and cyclopamine-treated limbs, and *Fgf8* was significantly downregulated in BMS compared to cyclopamine-treated limbs (Figures 1D,E). qRT-PCR for downstream targets of Fgf signaling, ETS transcription factor family genes *Etv1* and *Etv4* showed that *Etv4* was significantly downregulated in both the drug treatments (Figure 1E). These data supported that BMS was more effective at inhibiting Shh signaling compared to a max dose of cyclopamine. Although BMS drug treatment ultimately resulted in no proximodistal outgrowth, analysis of initial limb bud size and expression of direct downstream targets at stage 46 revealed that neither drug completely inhibited Shh signaling when treated at stage 39.

Although we used a max dosage of cyclopamine that embryos could tolerate without lethality (twice the concentrations used in previous amphibian studies), to rule out any potential for delayed activity of cyclopamine (compared to BMS), we exposed embryos to the two drugs prior to limb field formation (Figure 1F). Embryos treated at neural fold stage 19/20 with cyclopamine still developed a humerus and radius, whereas BMS completely inhibited limb formation (Figures 1F–H). These data demonstrate that BMS more completely inhibits the Shh signaling pathway compared to cyclopamine when used on salamander embryos and that Shh signaling regulates the earliest stages of axolotl limb development similar to phenotypes recovered from Shh inactivation during pectoral fin development in zebrafish (Neumann et al., 1999). In addition, the incomplete inhibition that we observed using cyclopamine allowed us to confirm that Shh also governs anteroposterior patterning across the limb mesoderm after bud outgrowth (Towers et al., 2008).

Differences across the drug treatments raised the possibility that previous cyclopamine studies may have overlooked an early role for Shh signaling in chick limbs. To test this idea, we inhibited Shh signaling during chick limb development using BMS beginning at stage HH14 to exclude the possibility of hedgehog pathway activation prior to the treatments (Figures 1H–J Supplementary Figures S2A–S2D). In contrast to our results in axolotls, chicken embryos treated with BMS developed limbs with a stunted stylopod, zeugopod, and autopod similar to the most severely affected pair of wings in a previous study using cyclopamine (Scherz et al., 2007) (Figure 1J, Supplementary Figures S2A–S2D). These findings substantiate that Shh signaling in amniote limbs functions primarily to pattern the anteroposterior axis where it acts subordinately to the AER which controls proximodistal outgrowth and skeletal differentiation (Figure 1H, Supplementary Figures S2B, S2D). Our findings in axolotls, however, show that Shh signaling first coordinates expansion of limb progenitor cells and proximodistal outgrowth of the limb bud in salamanders (Figure 1H).

### Axolotl *Shh* Crispants Completely Lack Forelimbs

**FIGURE 2:**
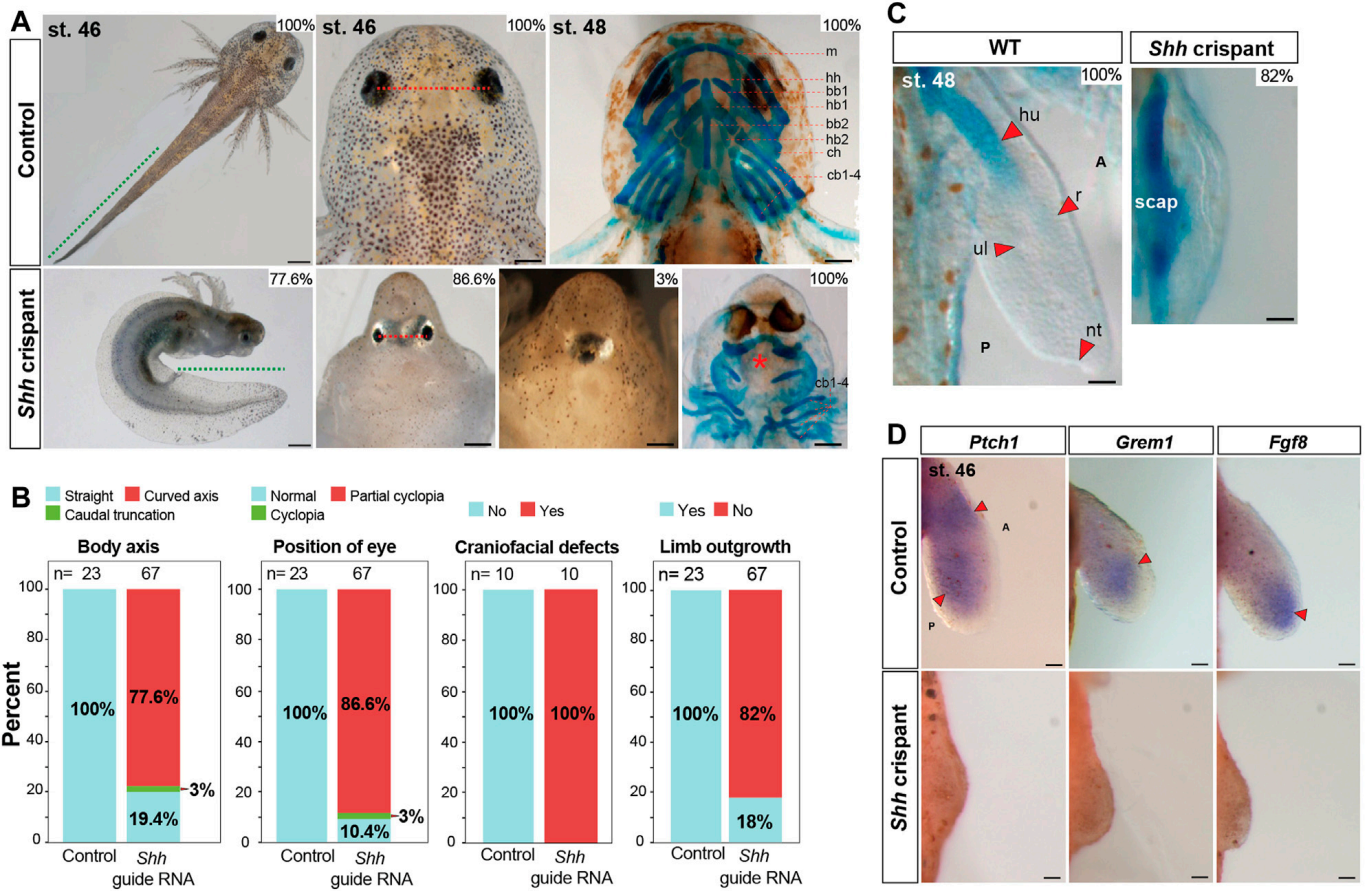
Axolotl *Shh* crispants completely lack forelimbs. **(A,B)** Phenotypes like body axis, position of the eye, and Alcian blue/Alizarin red staining of craniofacial structures of CRISPR control and *Shh* crispant larvae (*n* = 23 for CRISPR control and *n* = 67 for *Shh* crispant larvae for body axis and eye position analysis, *n* = 10 each for CRISPR control and *Shh* crispant larvae for Alcian blue/Alizarin red staining of craniofacial structures). Green dotted line, tail length; red dotted line, distance between the eyes; red asterisk, loss of anterior cranio-facial structures. Scale bar = 1 mm (for body axis) and 500 µm (for eye position and cranio-facial structures). **(B,C)** Alcian blue/Alizarin red staining for stage 48 limbs of CRISPR control and *Shh* crispant larvae (*n* = 10). Scale bar = 500 µm. **(D)** *In situ* hybridization for genes *Ptch1*, *Grem1*, and *Fgf8* in CRISPR control and *Shh* crispant larvae at stage 46 limbs (*n* = 3 or 4 per treatments). Red arrows, expression domains. Scale bar = 100 µm. m, meckel; hh, hypohyale; bb1, basibranchial 1; hb1, hypobranchial 1; bb2, basibranchial 2; hb2, hypobranchial 2; ch, ceratohyal; and 4 cb1-4, ceratobranchials.

To further interrogate the function of Shh signaling during salamander limb development, we genetically inactivated *Shh* in fertilized axolotl zygotes using CRISPR/Cas9. By designing three complementary guide RNAs to the *Shh* locus and separately injecting these into fertilized single cells, we recovered overlapping and robust mutant phenotypes using all three guide RNAs, thereby ruling out the chances of off-target effects (Figures 2A,B and Supplementary Figures S3A, Supplementary Figures S4). Next-generation sequencing (NGS) confirmed the efficiency of the three guide RNAs to create a frame mutation rate of ∼99% allowing us to analyze F0 larvae (Supplementary Figures S3B, Supplementary Table S2). Out of 300 embryos injected with either *Shh* guide RNA#1, 2, or 3, 67 survived and >80% of the F0 crispants that we screened presented a range of severe craniofacial defects, including partial to complete cyclopia, caudal truncations, and a curved body axis (Figures 2A,B); phenotypes that were similar to those observed in *Shh*-null mice (Chiang et al., 1996; Chiang et al., 2001). Although complete cyclopia occurred in relatively few F0 crispants (3%), 87% of the crispants exhibited eyes that were positioned with little to no interocular separation (Figures 2A,B). This resulted in a tight correlation between reduced interocular distance and the appearance of a bulge on the front of the head similar to the formation of a proboscis in *Shh*-null mice (Chiang et al., 1996). *Shh* mutants also had smaller heads and lacked most of the anterior craniofacial skeleton including jaws (Figure 2A).

In addition to these defects, almost all *Shh* crispants completely lacked forelimbs, a phenotype similar to zebrafish *Shh* mutants but in contrast to *Shh*-null mice, and chick embryos treated with BMS (Figure 1J and Figures 2B,C) (Chiang et al., 1996; Chiang et al., 2001; Neumann et al., 1999). Owing to a lack of jaw structures in the knockout animals that precluded them from eating, we analyzed the limb skeletons at stage 48 just prior to the onset of feeding (Figure 2C). At stage 48, 100% of control larvae showed a chondrifying humerus, radius, and ulna, whereas 82% of the *Shh* knockouts showed only elements of the pectoral girdle (Figure 2C). Using a previously published guide RNA against *Tyrosinase* (*Tyr*) (Fei et al., 2018), we observed loss of pigmentation in all injected animals, but, otherwise, normal embryos; a result that reinforced the specificity of CRISPR/Cas9 in axolotl embryos (Supplementary Figures S5A–S5E). Next, we analyzed the limb field area in *Shh* knockout animals at stage 46 where limb buds should form and observed almost no outgrowth over the condensing pectoral skeleton compared to elongate limb buds observed in wild-type animals (Figures 2C,D). This outgrowth defect was even more pronounced than in our BMS treatments with ectoderm almost directly covering the scapula. Last, we analyzed downstream Shh targets in the limb field of *Shh* crispants at stage 46. Although we observed strong expression of *Ptch1*, *Grem1*, and *Fgf8* in control limb buds, we were unable to detect expression for these target genes in the forelimb fields of *Shh* crispants (Figure 2D). Together with our BMS experiments, these results demonstrate that Shh signaling is required to stimulate expansion of forelimb bud progenitors and control proximodistal outgrowth of the limb bud.

### Embryonic Flank Tissue Outside the Limb Field is Refractory to Limb Outgrowth Signals in Axolotl Embryos

**FIGURE 3:**
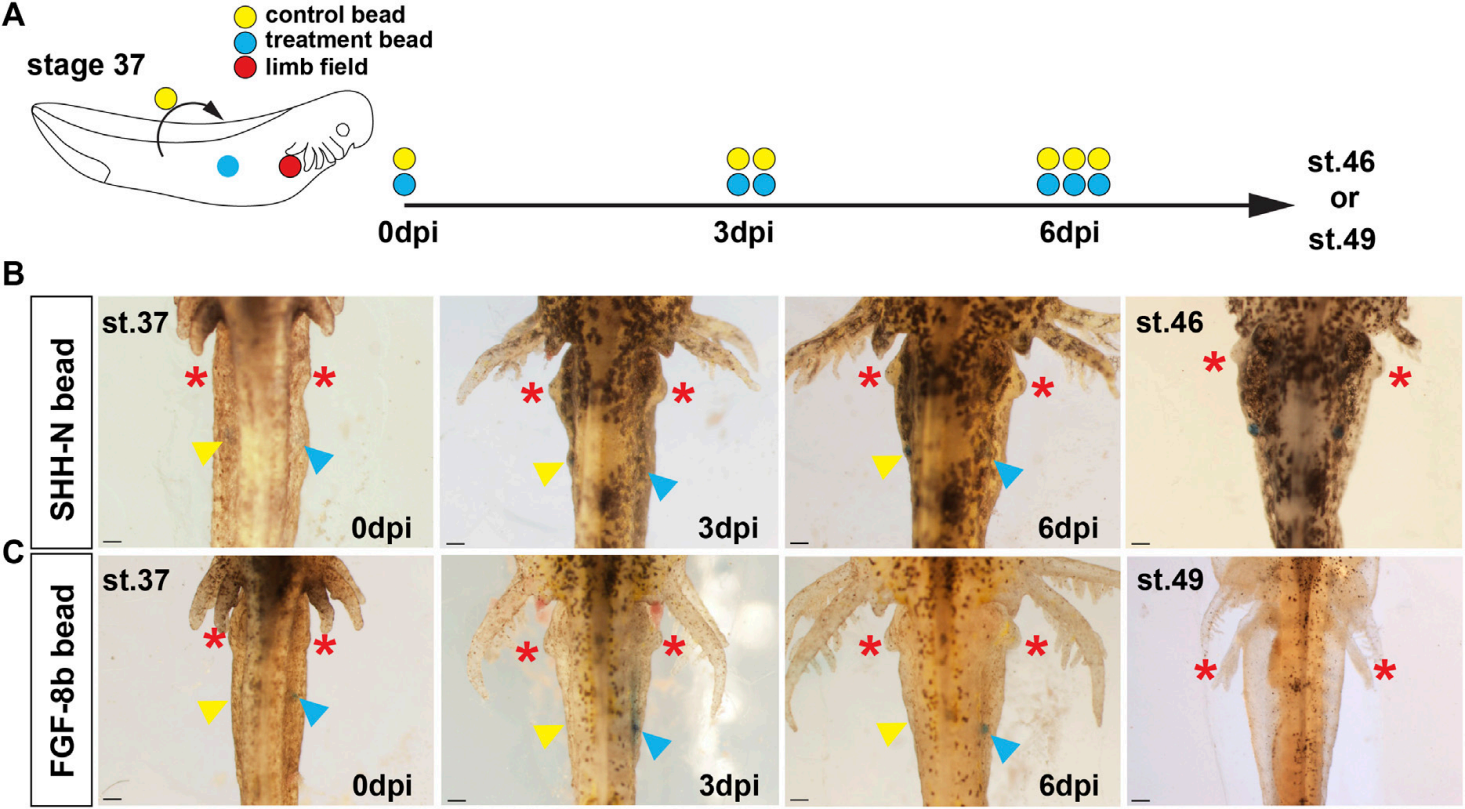
Stage 37 embryonic flank tissue in axolotls is refractive to exogenous SHH-N or FGF-8b proteins and does not form ectopic limb outgrowths. **(A)** Schematic representation of the embryonic bead implantation experiment using stage 37 axolotl embryos. Colored circles represent the right limb field (red), relative implantation site of protein-soaked beads (blue) or 1× PBS/0.1% BSA soaked beads (yellow). **(B,C)** SHH-N and FGF8 protein-soaked beads were grafted into the right flank lateral to the somites (blue arrow), and 1× PBS/0.1% BSA beads were implanted into symmetrical positions in the left flank (yellow arrow). Three days post first bead implantation (dpi), a second pair of beads were implanted into the same location as the first beads, and, consequently, a third pair were implanted at 6 dpi. Evidence of ectopic limb development was tracked until stage 46 (for SHH-N bead) or stage 49 (for FGF-8b beads), and, in either treatment condition, we did not observe evidence of limb outgrowth. Red asterisks mark emergence and growth of the normal forelimbs. Scale bar = 1 mm.

In chicken embryos, implantation of FGF protein is sufficient to induce a limb from uncommitted flank tissue supporting Fgf signaling at the apex of a molecular limb program that can induce a secondary limb field (Cohn et al., 1995). To ascertain whether Shh signaling could alone trigger limb bud outgrowth, we grafted SHH-N protein (in 1× PBS with 0.1% BSA) infused Affi-Gel beads into the right flank of stage 37–39 wild-type axolotl embryos, several days before forelimb buds emerge from the forelimb field and monitored for development of an extra limb bud at stage 46 (Figure 3A). On the contralateral (left) side of these embryos, we implanted 1× PBS with 0.1% BSA soaked control beads as control for the bead implant (Figure 3A). In either case, we did not observe the development of ectopic limb buds from the flank sites where we implanted SHH-N or control beads (Figure 3B).

In contrast to the flank sites with implanted beads, normal forelimb buds were evident at 3 days post first bead-implantation (dpi) (Figure 3B). This result suggested that flank mesoderm was already committed to a non-limb fate but did not rule out the possibility that another factor could induce an ectopic limb bud. Fgf8 is the endogenous inducer of chick limb formation and implanting FGF-8b soaked beads can induce an ectopic limb bud and development of a complete limb. Similar to our experiment with SHH-N, we did not observe ectopic forelimb buds in response to FGF-8b soaked beads (Figure 3C), and thus, these data supported that limb field progenitors are specified at precise positions very early during salamander development (Stocum and Fallon, 1982) and that neither SHH nor FGF8 could induce secondary limb fields in stage 37 embryos.

### *Sonic hedgehog* Controls Limb Bud Outgrowth in Axolotls

**FIGURE 4:**
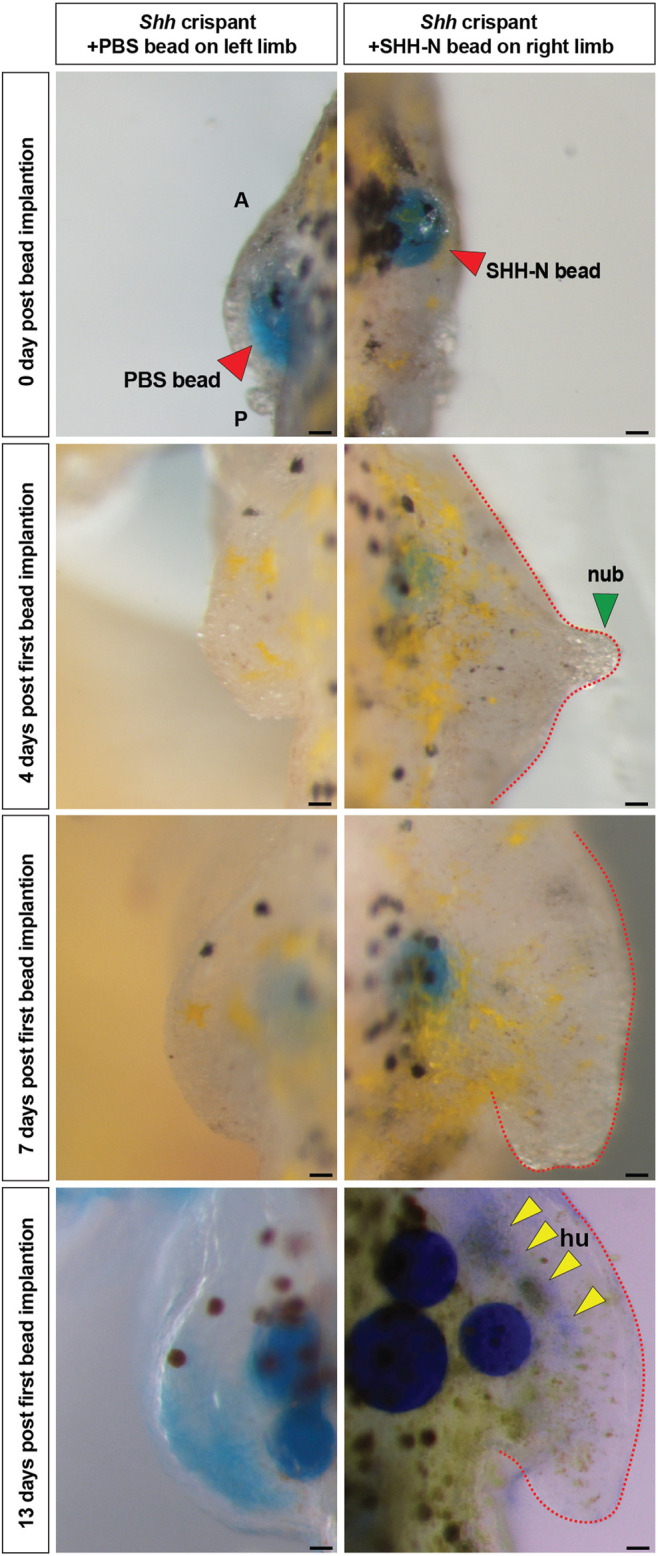
Exogenous SHH-N protein stimulates forelimb development from *Shh* crispant forelimb progenitors. Limb rescue experiments using beads loaded with SHH-N protein. Control Affi-Gel blue beads (red arrow) soaked in 1× PBS with 0.1% BSA and Affi-Gel blue beads (red arrow) soaked in SHH-N protein (0.5 or 0.25 µg/µl) in 1× PBS with 0.1% BSA were grafted into the left and right limb fields, respectively, of *Shh* crispants. Yellow arrows, faint Alcian blue staining for humerus. Scale bar = 100 µm. All the images are representative (with highest %) phenotypes. All limbs are projected in dorsal view with anterior “A” on top and posterior “P” on the bottom. hu, humerus; and nub, nubbin.

On the basis of these results, we next asked whether the forelimb field was specified in *Shh* crispants and, if so, whether implantation of SHH-N protein could induce forelimb formation from forelimb field progenitors. We implanted beads just prior to when limb buds would normally emerge, i.e., stage 39 (Figure 4). Stage 39 axolotls were characterized by longer and branched gills, distinct cloaca, pigmented eyes, and flanks, and these features were used to approximately stage in the control and *Shh* crispants (Schreckenberg and Jacobson, 1975). Affi-Gel blue beads infused with SHH-N protein were implanted into the position of the forelimb field (somites 3–5), whereas beads containing 1× PBS + 0.1% BSA were implanted into the left contralateral forelimb field as controls and beads were replaced once every 3–4 days. Although none of the beads on the control flank stimulated limb development, in seven of nine animals, limbs emerged in response to SHH-N (*n* = 2 showed nubbin like outgrowth, *n* = 3 showed progression to stage 45 limb bud, and *n* = 2 showed progression to stage 46/47 limb bud) (Figure 4). Five of the nine limbs that emerged past a nubbin and showed proximodistal outgrowth, three exhibited cartilage formation at the humerus level (Figure 4). Because of the inability of the crispants to feed, we could not take the limbs out far enough to determine whether the entire limb skeleton formed. However, these data do demonstrate that forelimb progenitor cells are competent to respond to exogenously delivered SHH protein that is sufficient to stimulate expansion of forelimb progenitor cells, formation of a forelimb bud, proximodistal outgrowth of the limb bud, and skeletal differentiation of the developing limb.

### *Sonic hedgehog* Controls Cell Proliferation and Cell Survival During Axolotl Limb Development

**FIGURE 5:**
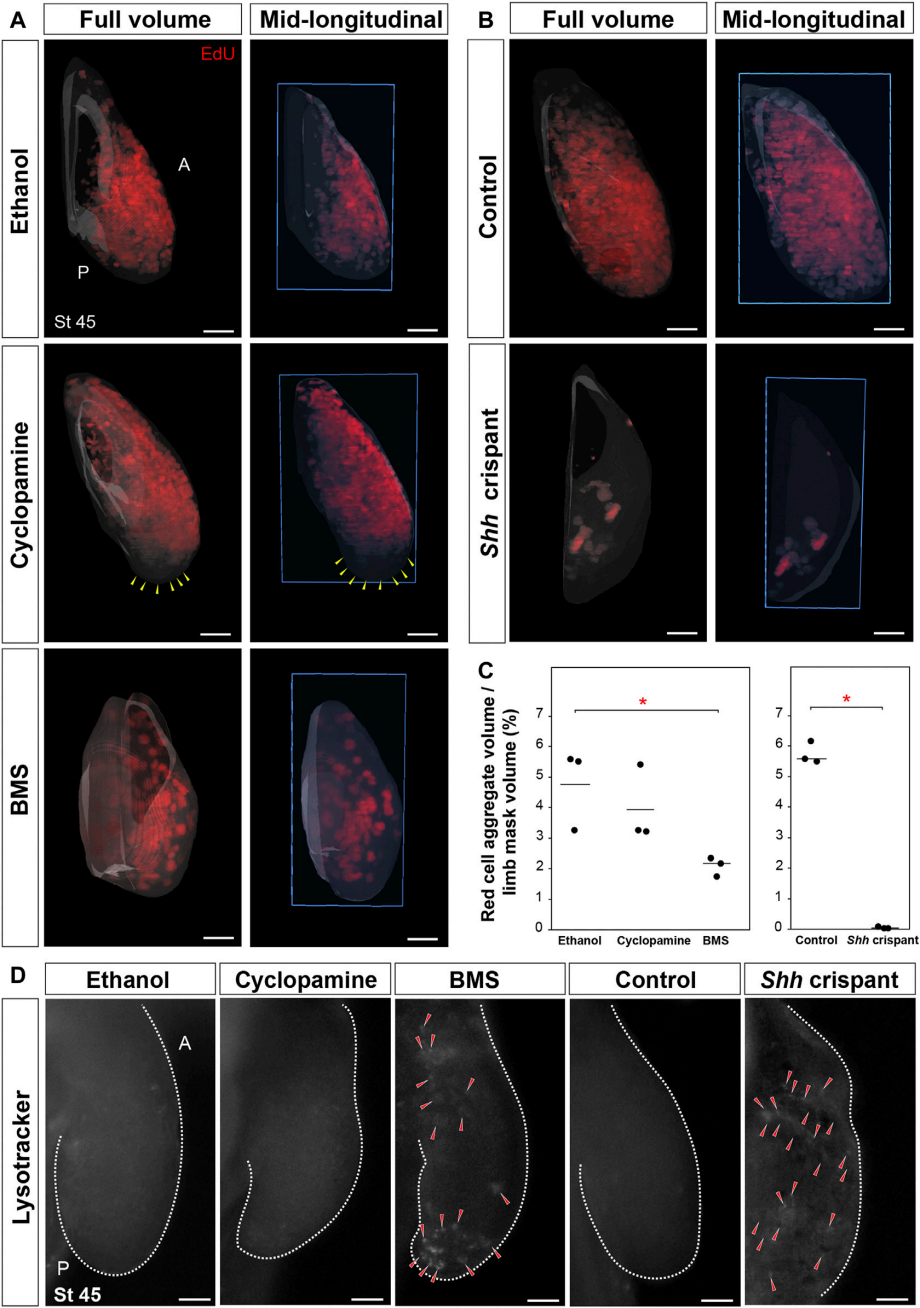
*Sonic hedgehog* controls cell proliferation and cell survival during axolotl limb development. **(A,B)** Light-sheet images depicting EdU-positive proliferating cells in stage 45 limbs from ethanol, cyclopamine, or BMS treatments and CRISPR control and *Shh* crispant larvae (*n* = 3 per treatment). Yellow arrows, zone lacking proliferating cells. Blue box, plane of mid-longitudinal section. **(C)** Stage 45 limbs from BMS-treated larvae and *Shh* crispant larvae showed a significant decrease in EdU-positive proliferating cells. Horizontal bars, mean values; asterisk, significant *p*-value [one-way ANOVA, Tukey–Kramer HSD post hoc test, *F* = 5.8, *p* = 0.038 (ethanol vs. BMS), *p* = 0.64 (ethanol vs. cyclopamine), and *p* = 0.12 (cyclopamine vs. BMS); *F* = 614, *p* < 0.0001 (control vs. *Shh* crispant); *n* = 3 per treatment]. **(D)** Cell death assay using LysoTracker in stage 45 limb in ethanol-, cyclopamine-, or BMS-treated larvae and CRISPR control and *Shh* crispant larvae (*n* = 3 per treatment). Red arrows, LysoTracker-positive cells. All limbs are projected in dorsal view with anterior “A” on top and posterior “P” on the bottom. Scale bar = 100 µm.

Although literature in chicken and mouse limbs show that AER–Fgf signaling regulates cell proliferation, cell survival, and limb outgrowth (Mariani et al., 2008; Summerbell, 1977; Janners and Searls, 1971), our BMS-treated and *Shh* crispant embryos suggested that Shh signaling regulated these processes in salamanders. To address this possibility, we quantified total cell proliferation in stage 45 limb buds across all treatment groups using light-sheet microscopy (Purushothaman et al., 2019). Compared to control limb buds, cyclopamine-treated limbs showed a decrease in proliferating cells only at the distal tip of the limb and did not show a significant decrease in total EdU+ cells (Figures 5A,C). In contrast, BMS-treated embryos and *Shh* crispant embryos showed a significant decrease among total EdU+ cells compared to control limbs (Figures 5A–C). Although the fraction of total proliferating cells as a function of total limb volume was not different between the cyclopamine and BMS treatments at stage 45, it was evident from the light-sheet images that the BMS-treated limbs were significantly smaller and contained fewer mesodermal cells (Figure 5A). *Shh* crispants had very few cells visible beneath the ectoderm because there was no outgrowth of the limb field (Figures 5B,C). Next, we analyzed cell survival using LysoTracker to label dying cells within developing stage 45 limbs (Mariani et al., 2008; Seifert et al., 2009). BMS-treated and *Shh* crispant larvae showed LysoTracker-positive cells throughout the limb buds, whereas none of the control larval limbs showed LysoTracker-positive cells (Figure 5D). Cell death was most prominent in the proximal and distal ends of BMS-treated limbs, whereas dying cells were present beneath the ectoderm on the flanks of *Shh* crispants (Figure 5D). Together, these results from *Shh* crispants and BMS-treated axolotl larvae support a model where Shh signaling seems to simultaneously control cell proliferation and cell survival in mesodermal progenitors of the limb field.

## Discussion

**FIGURE 6:**
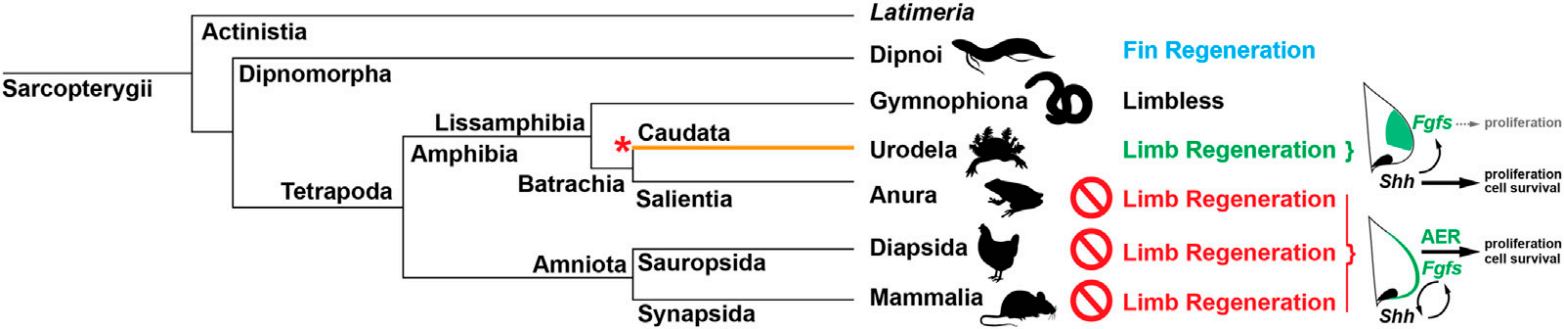
Limb regenerative ability may have evolved uniquely in caudates. Comparison of developmental mechanisms among tetrapods has revealed that urodeles possess a derived mode of limb development compared to anurans and amniotes including, absence of a morphological and molecular AER, mesenchymal restriction of canonical AER-*Fgfs*, absence of a positive feedback loop between Fgf and Shh signaling, and reliance on Shh-signaling to regulate proximal-distal outgrowth *via* cell proliferation and survival (orange line). Correlating with this unique mode of limb development, urodeles are the only extant tetrapods capable of complete limb regeneration at any post-embryonic life stage (larva, juvenile, or adult). One possible explanation for this tight correlation is that limb regenerative ability evolved within caudates as their mode of limb development was modified from the ancestral tetrapod state to reside within the mesenchymal tissue compartment (red asterisk). This opposes the hypothesis that limb and fin regenerative ability have a common origin among Sarcopterygii-with anurans and amniotes having lost limb regenerative ability (Nogueira et al., 2016). This hypothesis is based on common gene expression data derived from regeneration of cartilaginous elements in lungfish fins, a process similar to the regeneration of a cartilage spike in amputated *Xenopus* limbs.

Our study supports a model where molecular components present in pectoral fin and amniote forelimb buds are deployed uniquely during salamander limb development. Specifically, our results demonstrate that Shh signaling is essential for proliferation, survival, and expansion of forelimb field progenitor cells to form a salamander forelimb bud. As the limb bud emerges from the flank, Shh signaling stimulates *Fgf8* and downstream Fgf signaling targets in limb mesoderm, which support some cell proliferation at the distal tip of the limb bud but do not significantly contribute to proximodistal outgrowth of the limb (Purushothaman et al., 2019). Our results also uncovered that cyclopamine does not completely inhibit Shh signaling in salamanders when used at a maximum, non-lethal concentration for embryos and revealed that BMS is a more effective hedgehog inhibitor in salamander embryos. Genetic inactivation of *Shh* using CRISPR/Cas9 in newly fertilized zygotes confirmed our results using BMS and in combination with bead implantation experiments further revealed that Shh is not required to specify forelimb progenitor cells. Together, our data demonstrate that the molecular regulation of forelimb development has independently evolved in salamanders away from a reliance on reciprocal mesenchymal–epithelial Fgf signaling for limb bud outgrowth and proximodistal patterning: the arrangement present in anuran and amniote limbs and in actinopterygian pectoral fins (Figure 6).

Pectoral fin and forelimb development in actinopterygian and amniote embryos, respectively, rely on ectodermal–mesodermal cross-talk between Fgf- and Shh signaling. In amniote embryos where ZPA/AER cross-talk is present, proximodistal outgrowth of the limb bud is Shh-independent with Shh functioning as a cell survival factor, maintaining Fgf signaling in the AER, and coordinating anteroposterior patterning (Laufer et al., 1994; Niswander et al., 1994; Sanz-Ezquerro and Tickle, 2000; Kraus et al., 2001; Ros et al., 2003; Harfe et al., 2004; Scherz et al., 2007). Although Shh signaling does appear to regulate cell proliferation in chick embryos (Towers et al., 2008), it remains unknown whether this is an indirect effect mediated through Fgf signaling as occurs in zebrafish (Prykhozhij and Neumann, 2008)—a situation that is likely in light of our BMS results with chicken embryos. Proliferation of the fin/limb mesoderm and proximodistal expansion of the bud appears reliant on Fgf signaling from the AER, which also maintains Shh signaling from the ZPA (Lewandoski et al., 2000; Fischer et al., 2003; Nomura et al., 2006; Mariani et al., 2008). For instance, although zebrafish *shh* mutants completely lack pectoral fins, fin buds emerge normally, only later to regress as ectodermal Fgf signaling is lost (Neumann et al., 1999). Similarly, in *Shh* ^*−/−*^ mice, limb buds emerge smaller, but otherwise normally with an intact AER-expressing *Fgf8* (Chiang et al., 2001). However, without Shh signaling from the ZPA, the AER disappears, cell proliferation decreases, and posterior elements are lost (Chiang et al., 2001). It would appear that loss of *Shh* produces a different phenotype in fishes and amniotes (i.e., no pectoral fins versus elongate forelimbs with a humerus, radius, and single digit). However, the retention of skeletal elements in amniotes stems from the extent to which Fgf signaling is maintained in the absence of Shh signaling to sustain mesodermal proliferation and offset cell death reinforcing the reliance on Fgf signaling for fin and limb development in these groups. In fishes and amniotes, Fgf signaling is required for early expansion of fin/limb bud mesoderm where genetic disruption of AER-*Fgfs* leads to a complete absence of forelimbs/pectoral fins (Xu et al., 1998; Fischer et al., 2003; Nomura et al., 2006; Mariani et al., 2008). Thus, Fgf signaling from the ectoderm controls expansion of the fin/limb progenitor pool until such time that differentiation of the fin/limb skeleton begins.

In contrast to fishes and amniotes, our work demonstrates that ectodermal–mesodermal cross-talk disappeared in salamanders when Fgf signaling moved into the mesoderm (Purushothaman et al., 2019)—an event that also led to an erosion of the tight linkage between the two signaling pathways and an increased reliance on Shh signaling. Importantly, one consequence of this shift was that limb development in urodeles became largely independent of Fgf signaling. Inhibiting Fgf signaling in salamanders produces a relatively normal limb (minus a digit) that results from an overall small decrease in cell proliferation (Purushothaman et al., 2019), whereas inhibiting Shh signaling in this study resulted in a complete lack of forelimbs. Although our results using BMS or genetic inactivation of *Shh* make clear that limb bud cells cannot survive without Shh signaling, the emergence of small limb buds in response to BMS supports that a miniscule amount of Shh signaling is sufficient to stimulate cell proliferation among forelimb field progenitors—a result that could have manifested indirectly through residual Fgf signaling (Figure 1E). However, limb buds never emerged in our *Shh* crispants where *Shh* was genetically inactivated.

Implantation of SHH-N loaded beads into *Shh* crispants demonstrated that the molecular limb program in salamander limb progenitor cells is tuned to Shh signaling as a primary input. Thus, Shh signaling was sufficient to induce limb morphogenesis from forelimb progenitors in *Shh* crispants. In contrast, implantation of SHH-N beads (or FGF-8b beads) into flank mesoderm of wild-type embryos prior to when forelimb buds emerge could not induce ectopic limbs at stages 37–39. These experiments support that forelimb progenitors are specified much earlier during salamander development (Stocum and Fallon, 1982) and that these cells wait until endogenous *Shh* is activated, which, in turn, jumpstarts cell proliferation and activates downstream signaling pathways important for limb morphogenesis. Future studies examining molecular players that may lie upstream of *Shh* (e.g., RA and Tbx5/4) will provide important evidence as to which signals actually establish the field and secondarily induce *Shh* expression in these cells. Classical experiments in newts showed that pieces of otic placode implanted into the flank could induce an ectopic limb (Balinsky, 1925) and the otic placode expresses *Fgf8* among other factors. This raises the possibility that, although salamander limb development may have shifted its reliance on Fgf signaling for morphogenesis, it still may be a key factor for establishing the limb field.

Given the most recent phylogenetic models for relationships among extant tetrapods (Alexander Pyron and Wiens, 2011; Marjanović and Laurin, 2019), our findings support that urodeles possess a derived program for limb development relative to anurans and amniotes (Figure 6). This condition would also seem to deviate from the Shh-Fgf cross-talk that is required for fish pectoral fin development as discussed above. This molecular reorganization appears to occur after the establishment of limb field progenitors (Tulenko et al., 2013) as CRISPR/Cas9 knockout studies in newts show that upstream regulators of limb/fin bud initiation like *Tbx5* are conserved among vertebrates (Suzuki et al., 2018). The results of this study in combination with our previous results showing movement of Fgf signaling to the mesoderm (Purushothaman et al., 2019) and the work showing that the core long-range *Shh* enhancer (ZRS) is dispensable for limb development (Suzuki et al., 2018) support that the molecular circuitry during limb outgrowth has been reconfigured in urodeles. Interestingly, a study in medaka (*Oryzias latipes*) showed that, in addition to the canonical ZRS, a shadow ZRS (sZRS) controls *Shh* expression in fish (Letelier et al., 2018) and only deletion of both enhancers resulted in the complete loss of *Shh* expression and loss of pectoral fins (Letelier et al., 2018). Considered together, results from these studies point toward the existence of a shadow enhancer for *Shh* in urodele limbs as well. With the loss of a morphological and molecular AER (Purushothaman et al., 2019), the limb development program in urodeles relocated into the mesoderm with a role for the ectoderm diminished (Piatt and Kusner, 1960; Lauthier, 1985).

The derived nature of the urodele limb development program raises an intriguing hypothesis: the ability of adult cells to regenerate a functional limb may have evolved in concert with alterations governing the cellular and molecular control of limb development (Figure 6). In this scenario, adult limb regenerative ability emerged in urodeles after they diverged from anurans, a scenario contrary to the current view where adult limb regeneration was present in the tetrapod ancestor only to be lost in all the other major extant lineages (Fröbisch et al., 2014; Darnet et al., 2019a). Support for this new hypothesis comes from regeneration studies in several vertebrate models. First, experiments in salamanders and newts have shown that the regenerative capacity of blastema cells (the proliferative mass of mesenchymal cells at the amputation plane) lies within these cells in that they exhibit self-organizing properties allowing them to undergo morphogenesis even when transplanted to foreign sites (Stocum, 1984). Thus, they are reliant on signaling pathways that they themselves generate, not necessarily on reciprocal mesenchymal–epithelial signaling as seen during anuran and amniote limb development. Second, Shh signaling is required for blastemal cells to undergo regeneration, although functional studies using cyclopamine during normal limb regeneration in axolotls have yielded contradictory phenotypes not dissimilar from the disparity revealed between our cyclopamine and BMS treatments (Roy and Gardiner, 2002; Nacu et al., 2016). Although an earlier study showed that Shh signaling just controls anteroposterior patterning during limb regeneration (Roy and Gardiner, 2002), a later study using almost double the concentration of cyclopamine showed that Shh signaling was required for regeneration where it maintains *Fgf8* expression that is crucial for regenerative outgrowth (Nacu et al., 2016). Moreover, the canonical AER-specific *Fgfs* (*Fgf8*, *9*, and *17*) were re-expressed in the limb mesenchyme during normal limb regeneration. These results suggest Fgf signaling may play a more prominent role during regeneration where it functions in a positive feedback loop with Shh signaling (Nacu et al., 2016). Third, although *Xenopus* can regenerate limb buds and even parts of limbs during limb development (Dent, 1962), they cannot regenerate limbs following metamorphosis and instead generate unpatterned cartilage spikes. Limb regenerative ability is lost as the limb skeleton differentiates during development and is tied, in part, to an inability of blastemal cells to re-activate *Shh* and *Fgf10* (Yokoyama et al., 2000). *Shh* is not reactivated in the mesenchyme at regeneration-incompetent stages nor is ectodermal *Fgf8*. This echos the failure of chicken limb buds to regenerate where they are unable to restore an AER and Fgf signaling (Kostakopoulou et al., 1996). Although a recent study suggested that the loss of limb regenerative ability in *Xenopus* was due to an inability of limb cells to reprogram to a more developmental state (aka dedifferentiation) (Lin et al., 2021), a previous study in *Xenopus* showed that partially restoring limb regenerative ability required limb bud cells but only when those cells overexpressed ß-catenin and were exposed to SHH and FGF10 (Lin et al., 2013). These studies reinforce that blastemal cells require the capacity to activate self-organizing potential through the expression of self-sustaining signaling pathways and underscore the key role that patterning mechanisms play for regeneration to occur.

Although some investigators have argued on the basis of comparative gene expression data that an ancestral regeneration program exists for fins and limbs (Nogueira et al., 2016; Darnet et al., 2019b; Lu et al., 2019), these studies do not account for the fact that common gene expression profiles obscure functional relationships between signaling pathways. Many comparative studies also fail to account for interspecific differences in age or growth mode when assessing regeneration, two traits that likely regulate the availability of cells to participate in regeneration. As such, our results underscore the need to expand limb development studies across a more diverse array of vertebrates, especially anamniotes. For instance, although the relatively few molecular studies in anurans support a mode of limb development more aligned with amniotes, the data also suggest that an alternative dorsoventral patterning system may be in place (Christen and Slack, 1998). In addition, although anurans appear to exhibit conserved ectodermal–mesodermal cross-talk (Wang and Beck, 2014), studies in the direct-developing frog *Eleutherodactylus coqui* indicate that a morphological AER is not required for compartmentalized ectodermal *Fgf8* expression (Richardson et al., 1998; Gross et al., 2011). A recent study showing the absence of *Fgf8* during the development of bowfin pectoral fins with an AER further supports the plasticity in the limb/fin molecular program (Thompson et al., 2021). Relative to amniote limbs that develop with input from the somites, amphibian limbs exhibit delayed development and a high degree of self-organization in that transplantation of limb buds to other parts of the body produce a relatively normal limb (Stocum and Fallon, 1982), an ability which may help support limb regeneration (Galis et al., 2003). Although other tetrapods exhibit high degrees of self-organization in the limb field, our results offer that shifting the limb program entirely into the mesenchyme and toward a more pronounced reliance on Shh signaling to coordinate limb outgrowth may have permitted the self-organizing behavior of limb progenitors to execute patterning and, ultimately, functional regeneration.

## Materials and Methods

### Animal Husbandry and Tissue Harvest

Axolotls (*Ambystoma mexicanum*) (albino and wild type) were acquired from our own laboratory colony. Chicken eggs (University of Kentucky, Department of Animal Sciences) were incubated to required stages. All procedures were conducted in accordance with, and approved by, the University of Kentucky Institutional Animal Care and Use Committee (IACUC Protocol: 2013–1,174). For detailed methodology of animal husbandry and tissue harvest, refer to the work of Purushothaman et al. (2019).

Axolotl larvae were reared at 20°C–21°C and larvae used for drug treatments, Alcian blue/Alizarin red staining, whole mount *in situ* hybridizations, cell proliferation, and cell death assays were anesthetized using 1× benzocaine (Sigma) and fixed overnight in 4% paraformaldehyde (PFA) at 4°C. For qRT-PCR, larvae were anesthetized using 1× benzocaine, and limb tissue samples were snap-frozen and stored at −80°C until further use.

Chicken embryos (Single-Comb White Leghorn) were incubated in 1,502 Sportsman incubator, at 99.5°F, with 40%–50% humidity, harvested at HH30-HH32, fixed overnight in 4% PFA at 4°C, and processed for various downstream assays.

### Drug Treatments on Axolotl Larvae and Chick Embryos

Drug treatments on stage 39 axolotl larvae were done according to Purushothaman et al. (2019). Larvae were reared in six-well plates and kept in dark throughout the experiment. A working stock of cyclopamine (5 mg/ml; Sigma) and BMS (5 mg/ml; Cayman Chemical, Ann Arbor, MI, USA) was prepared in 100% ethanol and 0.6 µl from this stock was added into 3 ml of 20% Holtfreter’s solution per well (1 μg/ml final concentration). An equal amount of 100% ethanol (0.02%) was added into control wells. Higher concentrations tested for BMS were lethal. For earlier drug treatments, de-jellied axolotl embryos were treated with ethanol, cyclopamine, or BMS at neural fold stage (stage 19/20) for 10 days. The solutions were replenished every 2 days and treatments lasted for 10 days. For all drug experiments, survival rate (compared to controls), overall animal health (i.e., that they are feeding, swimming, no buoyancy issues, and well-formed gills with adequate blood supply), and body length were monitored. For the drug concentrations administered in this study, we found no significant difference for these monitoring criteria between treatment and control animals.

Drug treatments on chicken embryos were done at HH14/15. Five milliliters of albumen was removed from the bunt end using a 5-ml syringe and 22G1½ needle. The eggs were windowed, and vitelline membrane around the limb bud was removed. The embryos were treated with 5 µl of solution (1 mg/ml) of BMS in DMSO or DMSO (control) followed by 200 µl of Ringers solution with Pen-strep (100 U/ml). The window was covered with a scotch tape, and the eggs were reincubated until harvest at HH30/32.

### Alcian Blue and Alizarin Red Staining

Alcian blue and Alizarin red staining on axolotl larvae was done as previously described in Purushothaman et al. (2019). Fixed axolotl larvae were dehydrated in graded ethanol series and stained with 0.02% Alcian blue 8GX (Sigma Aldrich, St. Louis, MO, USA) in 70% ethanol and 30% glacial acetic acid for 3 h to overnight. Larvae were rehydrated in graded ethanol series and then stained with 0.1% Alizarin red (Sigma Aldrich, St. Louis, MO, USA) in 1%KOH overnight. Larvae were cleared in 1%KOH/glycerol series: 3KOH:1glycerol (imaged when cleared), 1KOH:1glycerol (1 day) and 1KOH:3glycerol (stored at room temperature). A subset of 10 larvae each were used from 23 (control) and 67 (*Shh* guide RNA injected) larvae for Alcian blue and Alizarin red staining to analyze craniofacial defects and skeletal elements within the limbs.

Chicken embryos were harvested at HH30/32 and fixed in 100% ethanol for 2 days, stained with 0.1% Alcian blue 8GX (Sigma Aldrich) in 80% ethanol/20% acetic acid for 1 day, and cleared in 1% KOH before imaging.

### Whole Mount *In Situ* Hybridization

Sense and antisense probes for *Ptch1*, *Grem1*, and *Fgf8* axolotl genes were synthesized according to Purushothaman et al. (2019). Fixed axolotl larvae were dehydrated in graded methanol/PBT series stored in 100% methanol at −20°C until further use. Larvae were rehydrated in a graded methanol/PBT series and bleached with 6% H_2_O_2_/1× PBS for 1 h under ice-cold conditions. Larvae were permeabilized with Proteinase K (20 μg/ml; Roche) in 1× PBS for 7–10 min, fixed with 0.2% gluteraldehyde/4% PFA at room temperature, and incubated overnight in hybridization buffer [5% Dextran sulphate, 2% blocking powder from Roche, 5X SSC, 0.1% TritonX, 0.1% CHAPS from Sigma Aldrich, 50% formamide, tRNA (1 mg/ml) from Roche, 5 mM EDTA from Sigma, and Heparin (50 μg/ml) from Sigma] at 60°C. The tubes were replaced with fresh hybridization buffer, 0.1–1 µg of probe was added into each vial and incubated at 60°C for 2 days. High stringency washes were done with 2X SSC/0.1% CHAPS thrice for 20 min each, 0.2X SSC/0.1% CHAPS four times for 25 min each and with KTBT [15 mM Tris-HCl (pH 7.5), 150 mM NaCl, 10 mM KCl, and 1% Tween 20] twice for 5 min each. Larvae were blocked with 20% goat serum in KTBT for 3 h, later treated with fresh blocking solution with an anti-Digoxigenin-AP, Fab fragment antibody (Roche) at 1:3,000 dilution, and incubated overnight at 4°C. Larvae were washed with KTBT five times for 1 h each and then incubated in KTBT overnight at 4°C. Larvae were washed with NTMT [100 mM Tris-HCl (pH 9.5), 50 mM MgCl_2_, 100 mM NaCl, and 1% Tween 20] and incubated in NBT/BCIP (Roche) solution (BCIP, 0.17 mg/ml; NBT, 0.33 mg/ml; 10% DMF in NTMT) until a signal developed with minimum background staining.

### qRT-PCR Analysis

Stage 39 axolotl larvae were reared in six-well plates in either 0.02% ethanol, cyclopamine (1 μg/ml), or BMS (1 μg/ml) until stage 46. Whole limbs were dissected from the body wall, immediately snap-frozen, and stored at −80°C until RNA extraction. *n* = 3 was used for each condition and each replicate represented a pool of limbs (both left and right) from 10 to 20 animals.

RNA was extracted using TRIzol reagent (Invitrogen), cDNA was synthesized from 0.5 to 1 µg of RNA using SensiFast cDNA synthesis kit, and qRT-PCR was performed using iTaq Universal SYBR Green Supermix (Biorad) (refer Supplementary Table S1 for primer sequences). Melting curve was analyzed to confirm primer specificity.

*Rlp32* were used as the internal control/house-keeping genes for the experiments, respectively, since there was no significant fold change in the 2^−Ct^ values (Schmittgen and Livak, 2008). 2^−ΔΔCt^ method was used to calculate the fold change values between control (ethanol) and treatment (cyclopamine or BMS) groups (Schmittgen and Livak, 2008).

### Guide RNA Synthesis

Protocol for guide RNA synthesis was partially adapted from the work of Fei et al. (2018). The mRNA coding sequence for *Shh* gene was accessed from https://www.axolotl-omics.org/search. DNA template oligos for guide RNA synthesis were designed using cloud-based informatics platform Benchling and oligos (20-mer or 18-mer) with high on-target and off-target scores were selected. Three DNA template oligos for separate *Shh* guide RNAs were ordered from IDT with a 5′ adapter and T7 promoter at the 5′ end and a 3′ overhang sequence complementary to the constant sequence at the 3′ end [refer to the work of Fei et al. (2018) for schematic diagram and Supplementary Table S1 for sequences]. The DNA template oligo for *Tyrosinase* (*Tyr*) guide RNA ordered from with 5′ adapter, T7 promoter sequence, GG nucleotides at the 5′ end, and a 3′ overhang sequence complementary to the constant sequence at the 3′ end [refer to the work of Fei et al. (2018) for schematic diagram and Supplementary Table S1 for sequences]. The DNA template oligo for *Tyr* guide RNA was adapted from the work of Fei et al. (2018).

The DNA template oligo was amplified using the Phusion DNA polymerase kit (NEB, cat# M0530S). Refer to Table S1 for primer sequences. The reaction mix and PCR reaction were as follows:

**Table:** 

Phusion GC buffer, 5x	10 µl
dNTPs (10 mM)	0.5 µl
gRNA-fw2 (100 µM)	0.35 µl
DR274-rev (100 µM)	0.35 µl
gRNA oligo (100 µM)	0.25 µl
Constant sequence (100 µM)	0.02 µl
Phusion DNA polymerase (2 U/µl)	0.5 µl
Nuclease-free water	38.03 µl
	50 µl

PCR Reaction.

**Table:** 

98°C	30 s	×1
98°C	20 s	×35
60°C	20 s	
72°C	20 s	
72°C	5 min	×1
4 °C	forever	×1

The PCR product was checked on a 1% agarose gel for single bands, purified (in 20 µl of water) using QIAquick PCR purification kit, and quantified. The purified product was used for guide RNA synthesis (*in vitro* transcription) using Ambion MegaShortscript Kit T7 (cat#AM1354). The *in vitro* transcription step was as follows:

**Table:** 

T7 10X reaction buffer	2 µl
T7 ATP solution (75 mM)	2 µl
T7 CTP solution (75 mM)	2 µl
T7 GTP solution (75 mM)	2 µl
T7 UTP solution (75 mM)	2 µl
Template DNA from previous PCR (500–750 ng)	xµl
T7 enzyme mix	2 µl
Water	make up to 20 µl

The reaction mix is incubated at 37°C overnight, and guide RNAs were precipitated by phenol/chloroform method as follows: the reaction mix was transferred into a 2-ml vial, 115 µl of water, and 15 µl of Ammonium acetate stop solution (from Ambion MegaShortscript Kit T7) and 500 µl of phenol + 500 µl chloroform mix were added, mixed until an emulsion formed, and centrifuged at 13,000 rpm for 1 min; aqueous phase was transferred into a fresh 1.5-ml vial; 2 volumes of ethanol was added and mixed well, chilled at −20°C for 15 min, and centrifuged at 13,000 rpm for 15 min; supernatant was carefully discarded; tubes were allowed to dry under the hood; and the RNA pellet was resuspended in ∼20 µl of water. The integrity of the guide RNAs was checked on a gel, quantified, and stored in −70°C (as 2 µl of aliquots) until microinjections.

### Microinjections

Protocol for microinjection was partially adapted from the work of Fei et al. (2018). The following injection mix was freshly prepared once the female started laying eggs:

**Table:** 

CAS9-NLS protein (PNA Bio, cat# CP03), reconstituted in 1× CAS9 buffer to 5 mg/ml	1 μl
Guide RNA (4 μg)	x μl
CAS9 buffer, 10× (200 mM HEPES, 1.5 M KCl, pH 7.5)	0.9 μl
Nuclease-free water	make up to 10 µl

The glass capillary tubing (OD = 1 mm, ID = 0.58 mm, length = 7.5 cm) was pulled (Sutter instruments Co.; settings: heat, 600; pull, 50; vel, 120; time, 165), and the injection mix was loaded into it. The needle tip was calibrated using a stage micrometer so as to inject a volume of 5 nl into each fertilized egg. For control injections (CRISPR control), injection mix minus guide RNA was injected into each single-cell fertilized egg.

The single-cell fertilized eggs were sterilized using 70% ethanol for 20 s, rinsed and dejellied in 1× MMR/Pen-strep solution, and transferred to 1× MMR/Pen-strep + 20% Ficoll solution for microinjections. Post injection, the eggs were transferred into fresh 1× MMR/Pen-strep + 20% Ficoll for 2 h and then transferred into 0.1× MMR/Pen-strep + 5% Ficoll for 24 h at 18°C. The healthy embryos were transferred into 24-well plates with 0.1× MMR/Pen-strep solution and not disturbed for 7 days. Fresh 0.1× MMR + Pen-strep solution was added on the eighth day, and solution was replenished once in 2 days until final harvest.

### Genotyping

Protocol for genotyping was partially adapted from the work of Fei et al. (2018). For genomic DNA extraction, 1-mm tail clips from CRISPR control and guide RNA–injected larvae were snap-frozen in 1.5-ml vials and stored at −80°C until further use. Later, in to the 1.5-ml vials, 100 μl of 50 mM NaOH was added and incubated at 95°C for 20 min and 10 μl of 1 M Tris (pH 7.5) was added, mixed well, spun, and quantified. The following PCR was performed to amplify the gene locus:

**Table:** 

Genomic DNA (200 ng)	xμl
Taq reaction buffer, 10×	2 μl
dNTPs (10 mM)	0.3 μl
Genotyping primer forward (10 μM)	1 μl
Genotyping primer reverse (10 μM)	1 μl
Taq DNA polymerase (5 U/μl)	0.1 μl
Nuclease-free water	make up to 20 µl

(Refer to Supplementary Table S1 for genotyping primer sequences).

PCR reaction.

**Table:** 

94°C	2 min	×1
94°C	30 s	×35
50°C, 60°C, or 70°C	30 s	
72°C	30 s	
4°C	forever	×1

The PCR product was checked on gel to verify single bands, quantified, and sent out for NGS (Amplicon-EZ, Genewiz) to sequence each gene locus.

### Bead Experiments

Axolotl larvae were used at developmental stage 37 (Schreckenberg and Jacobson, 1975) and reared in 24-well plates in 0.1× MMR/Pen-strep solution. Before any procedure, larvae were anesthetized using 1× benzocaine (Sigma), placed on 1% agarose plates with 1× PBS/0.1% BSA solution, and imaged under a stereoscope microscope. When majority larvae reached between stages 46–49, all larvae were anesthetized using 1× benzocaine, fixed in 4% PFA, washed with 1× PBS, and imaged under a stereoscope microscope.

SHH-N protein (R&D Systems, cat#1845-SH-025) was reconstituted in 1× PBS with 0.1% BSA. Each Affi-bead (Affi-Gel Blue Media, cat#153-7301, 150–300 μm) was incubated in 1 μl (0.25 or 0.5 μg/μl) of SHH-N protein for 2 h at room temperature or overnight at 4°C. FGF-8b protein (R&D Systems, cat#423-F8-025) was reconstituted in 1× PBS with 0.1% BSA. Each heparin/agarose bead (Sigma-Aldrich, cat#H6508) was washed in 1× PBS and incubated in 2 µl (0.5 μg/μl) of FGF8 protein for 2 h at room temperature. Prior to grafting, FGF8-soaked beads were transferred into 2 µl of 0.1% fast green dissolved in water to visualize the beads. Control beads were incubated in 1 μl of 1× PBS with 0.1% BSA for 2 h at room temperature or overnight at 4°C.

To test the limb forming potential of the presumptive trunk in wild-type larvae, a tungsten wire was used to make an incision into the posterior ectoderm where the control and treated (SHH-N or FGF8) beads were slid into the presumptive trunk on the left and right sides of the larvae, respectively. The beads were grafted when larvae reached stage 37. The same procedure was used after 3 and 6 days to implant a second and third bead following the first insertion. Live larvae were imaged at 0, 3, and 6 dpi (days post initial bead insertion). Experimental larvae were harvested once larval limbs reached stage 46/49.

Limb rescue bead experiments were done on larvae injected with either *Shh* guide RNA#3 or *Shh* guide RNA#1, 2, and 3 with evident axolotl *Shh* crispant phenotypes like curved body axis, partial to complete cyclopia, and no limb outgrowth. The SHH-N beads were grafted when the CRISPR control larvae reached stage 37. A nick was made at the approximate limb field position using a tungsten needle, and beads were grafted securely into the nicks. Protein-soaked bead was grafted into the right limb field, and control bead was grafted into the left limb field. Beads were replaced once in 3–4 days and experimental larvae were harvested once CRISPR control larval limbs reached stage 46/48.

### Cell Death and Cell Proliferation Assays

Refer to the work of Purushothaman et al. (2019) for detailed protocols of cell proliferation and cell death assays in axolotls. Post-hatch larvae were reared in six-well plates in 3 ml of either of the solutions: 0.02% ethanol, cyclopamine (1 μg/ml), or BMS (1 μg/ml) in 20% Holtfreters solution. CRISPR control and *Shh* crispants were reared in six-well plates in 3 ml of 0.1× MMR/Pen-strep solution.

For cell proliferation assay, larvae were additionally treated with EdU (0.1 mg/ml) for 24 h when control larvae (ethanol-treated or CRISPR control) reached stage 45, fixed overnight in 4% PFA, dehydrated in 1× PBS/methanol series, and stored in 100% methanol at −20°C until further use. Larvae were rehydrated backward through graded methanol series starting at 100% methanol and ending at 100% 1× PBS, treated with 2.5% trypsin (Gibco) for 10 min, permeabilized with proteinase K (20 μg/ml) in PBT for 7–10 min, fixed in 100% acetone at −20°C for 10 min, incubated in fresh click reaction solution [1× TRIS buffer saline, 4 mM CuSO_4_ in 1× TRIS buffer saline, 2 µl of Alexa-flour-594 Azide (Life technologies), 1 mM sodium ascorbate in 1× TRIS buffer saline] for 30 min on a rocker in the dark, incubated in DAPI (1:1,000 dilution) for 30 min, checked for fluorescence under a stereomicroscope, and stored at 4°C in the dark until light-sheet imaging.

For cell death assay, larvae were transferred into 24-well plates and treated with 200 µl of 5 µM LysoTracker Red DND-99 (molecular probes) in Hanks’ BSS for 45 min to 1 h at 20°C–21°C, fixed overnight in 4% PFA, dehydrated through a graded methanol/Hanks’ BSS series and stored in 100% methanol at −20°C until imaging.

### Microscopy and Image Analysis

Whole mount images of limbs for Alcian blue/Alizarin red staining, limb size measurements, *in situ* hybridization, bead experiments, cell death assays, body axis analysis, and eye position and pigmentation analysis were taken on an SZX10 light microscope (Olympus, Tokyo, Japan) using a DP73 CCD camera (Olympus). The microscope was equipped with CellSense software (CellSense version 1.12, Olympus Corporation).

EdU-stained stage 45 larval limbs were imaged using a Zeiss Lightsheet Z.1 (College of Arts and Science Imaging Center, University of Kentucky). Refer to the work of Purushothaman et al. (2019) for detailed protocol of light-sheet microscopy for axolotl limb buds. Zen software (Zeiss) was used for imaging, and samples were excited using 561- and 488-nm lasers. Arivis vision4D software (Arivis) was used for image processing. For total limb volume calculations, an object mask was hand drawn at each z-plane on the basis of the DAPI signal to outline the limb. Red cell aggregate volume and total limb volume were calculated using the previously standardized protocols, and volume values in cubic micrometers and voxel counts were given as outputs.

### Fiji Analysis

Melanocyte pigmentation in the eyes of stage 46 tyrosinase crispants and control larvae was measured using Fiji software (NIH) after calibrations. Eye pigmentation was measured as pixel intensity and the ranges were as follows: pixel intensity = 221 to 148 (high), 147 to 74 (moderate), and 73 to 0 (low).

### Statistics

All statistical analyses were performed using JMP (version Pro 12.10, SAS Institute Inc.) and Microsoft Excel. Bar and pie diagrams were made using Microsoft Excel.

For limb size between ethanol, cyclopamine, and BMS, one-way ANOVA followed by Tukey–Kramer HSD post hoc test was performed. Differences were considered significant if *p* < 0.05.

For qRT-PCR data, the 2^−ΔΔCt^ method was used to calculate fold changes of genes between ethanol, cyclopamine, and BMS groups. Calculations for mean Ct values, ΔCt values for treatment and control groups, ΔΔCt values between treatment and control groups, 2^−Ct^ and 2^−ΔΔCt^ fold change values were done using Microsoft Excel. One-way ANOVA followed by Tukey–Kramer HSD post hoc test was performed. Differences were considered significant if *p* < 0.05.

For light-sheet data, red cell aggregate volume/limb volume (%) was calculated in Microsoft Excel (red cells = EdU-positive cells). Post arcsin conversion, comparisons between control and treatment groups (ethanol vs. cyclopamine vs. BMS and CRISPR control vs. *Shh* crispant) were done by one-way ANOVA followed by Tukey–Kramer HSD post hoc test.

## Data Availability

The original contributions presented in the study are included in the article/Supplementary Materials, further inquiries can be directed to the corresponding author.
