# Supplementary material for: Sonic hedgehog is Essential for Proximal-Distal Outgrowth of the Limb Bud in Salamanders
Source: Front Cell Dev Biol. 2022 Apr 1;10:797352. doi: 10.3389/fcell.2022.797352 (PMC9010949; doi:10.3389/fcell.2022.797352)
Supplement: Supplementary file 1 [file DataSheet1.PDF]

Supplementary Material

Supplementary Figures

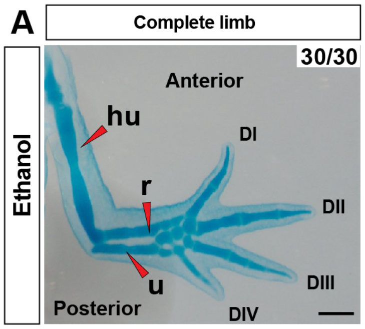

D

Treatment	n=	Humerus	Radius/ulna	# Digits	# cases	%
Ethanol	30	**Yes**	**Separate**	**4**	**30/30**	**100**
Cyclopamine	24	Yes	None	0	2/24	8
		Yes	?	0	1/24	4.2
		**Yes**	**radius**	**1**	**6/24**	**25**
		Yes	radius	1?	4/24	16.7
		**Yes**	**radius**	**2**	**6/24**	**25**
		Yes	radius	2	3/24	12.5
		Yes	radius	3	2/24	8.3
BMS	36	Yes	radius	1?	1/36	2.8
		Yes	radius	0	2/36	5.5
		**Bud**	**None**	**0**	**24/36**	**66.7**
		No	None	0	9/36	25

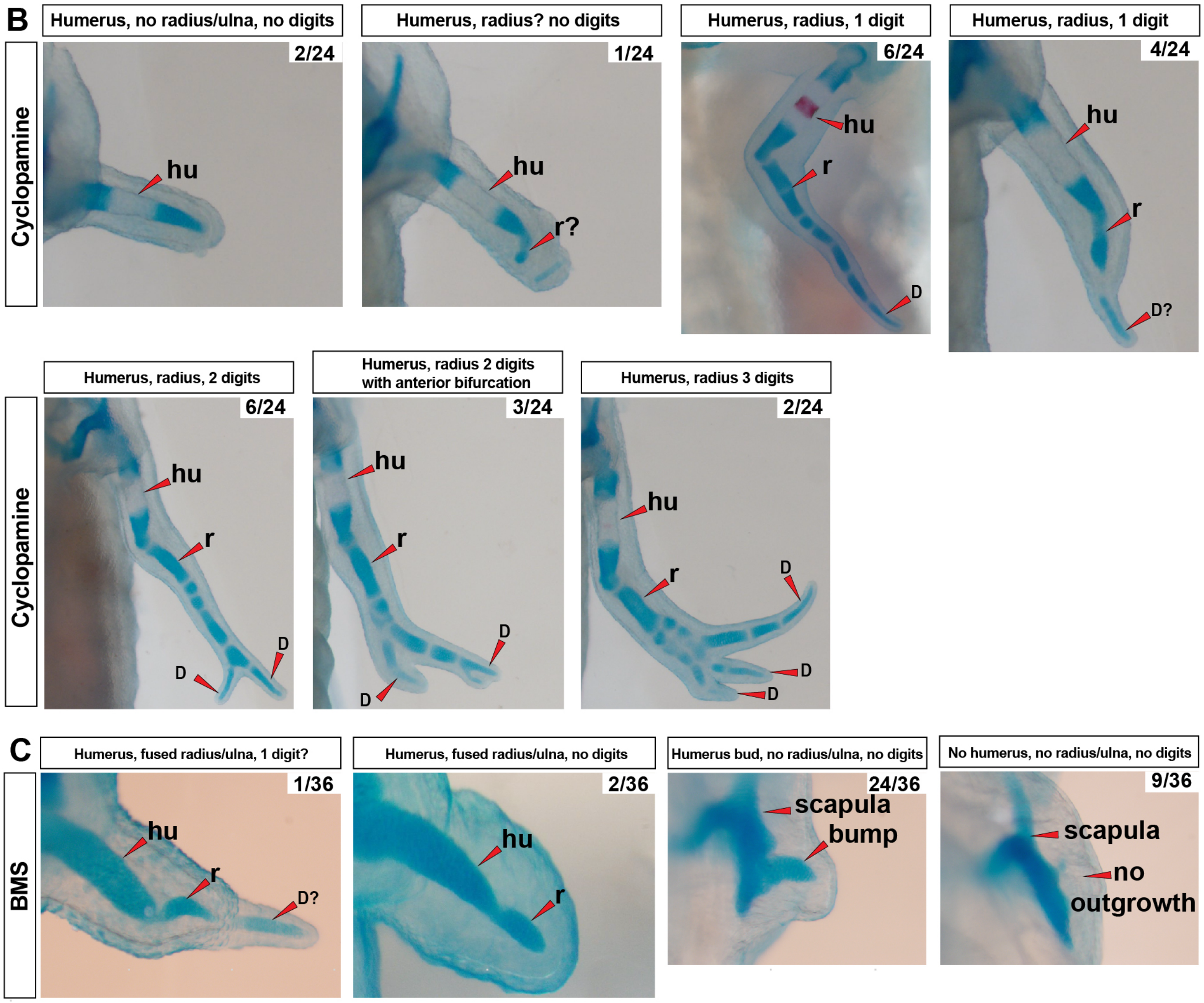

Figure S1. Detailed range of axolotl limb phenotypes associated with ethanol, cyclopamine and BMS-833923 treatments. (A-C) Alcian blue/Alizarin red staining for different limb phenotypes with case numbers in ethanol, cyclopamine and BMS treated larvae (limb n=30 for ethanol, =24 for cyclopamine and =36 for BMS). Scale bar=500 µm. **(D)** Table depicting treatments, limb phenotypes and percentage cases. hu, humerus; r, radius; ul, ulna and D, digits.

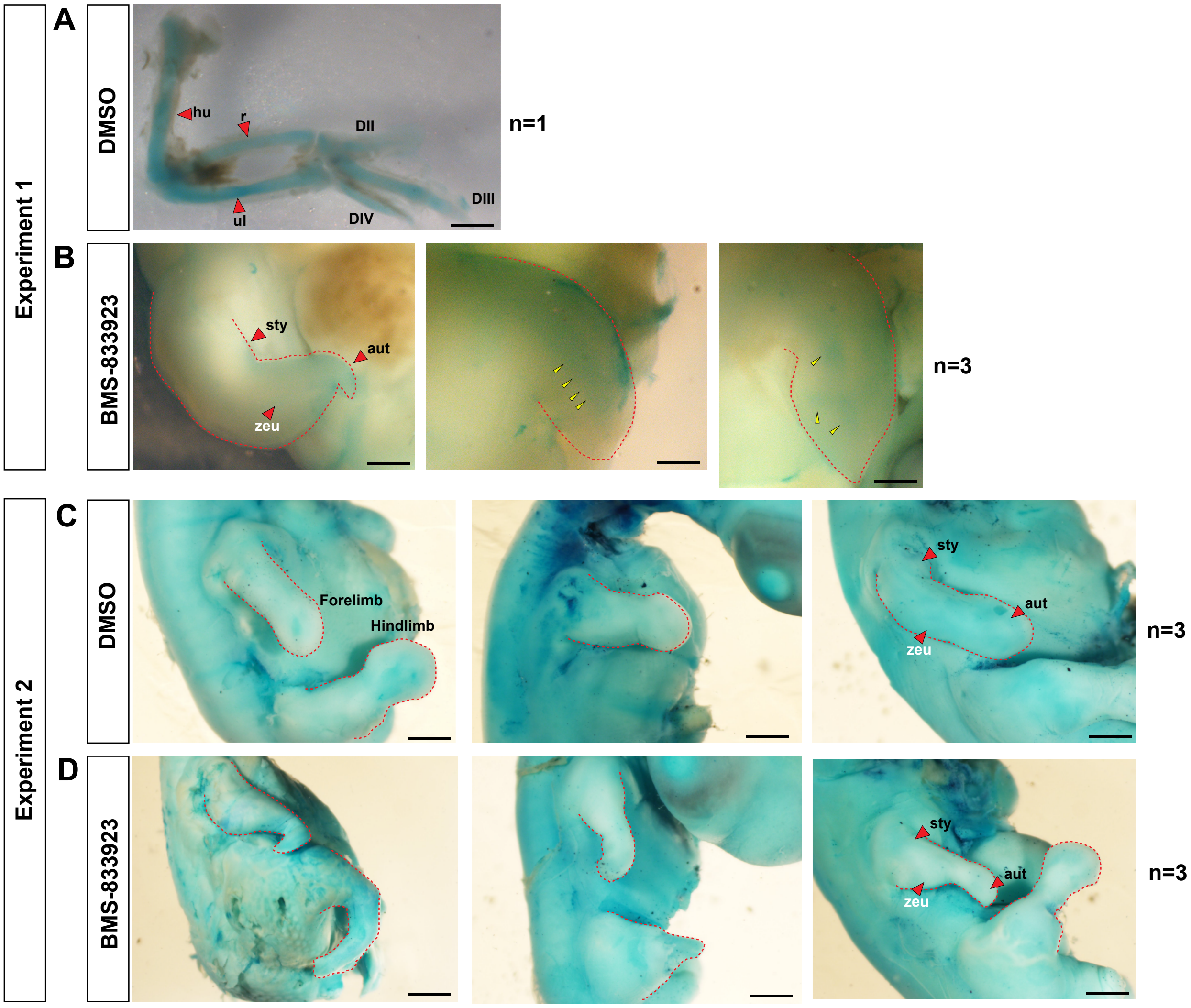

Figure S2. Detailed range of chicken limb phenotypes associated with DMSO and BMS-833923 treatments. (A and B) Alcian blue staining for different limb phenotypes with n numbers in DMSO and BMS treated larvae for 1st set of experiments. Scale bar=500 µm. Yellow arrows point towards faint Alcian blue staining (B). **(C and D)** Limb phenotypes with n numbers in DMSO and BMS treated larvae for 2st set of experiments. Scale=1mm. hu, humerus; r, radius; ul, ulna; D, digits; sty, stylopod; zeu, zeugopod and aut, autopod.

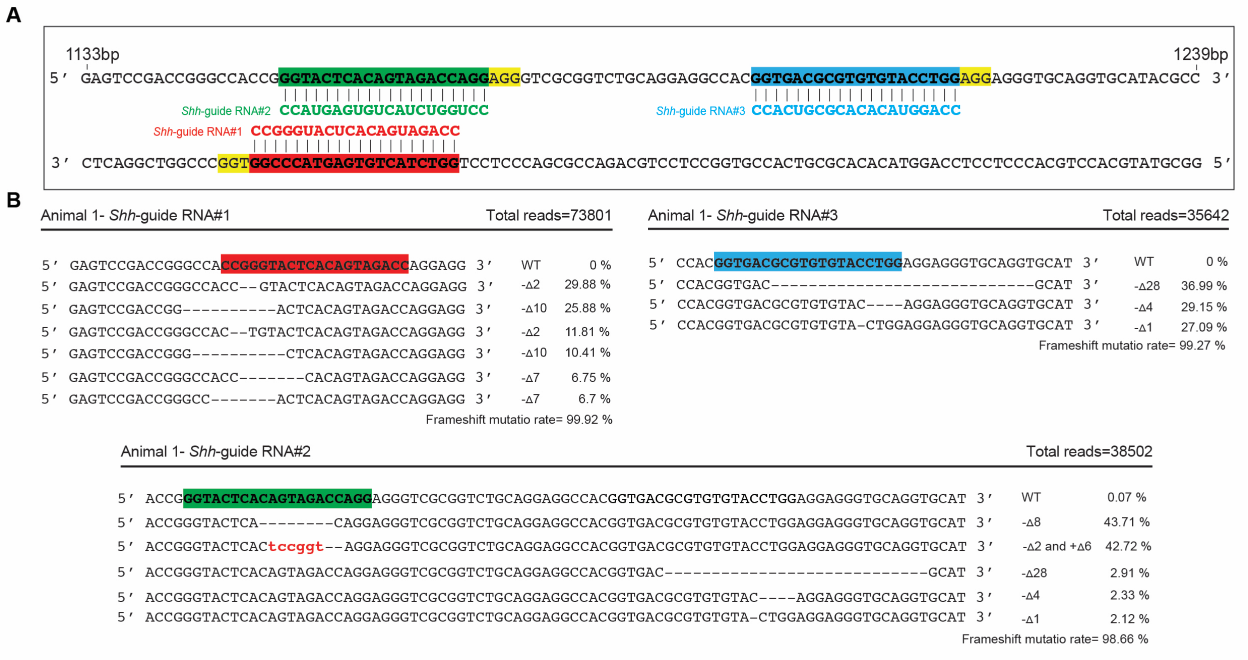

Figure S3. *Shh* guide RNA gene targets and NGS results of *Shh* crispants. (A) Target sequences for axolotl *Shh* (bold and highlighted in red, green and blue) and 20-mer *Shh*-guide RNAs #1 (red text), #2 (green text) and #3 (blue text) complementary to the target sequences. Protospacer Adjacent Motif or PAM (NGG) for each guide RNA target is highlighted in yellow. **(B)** Genotype of *Shh*-guide RNA injected larvae analyzed using AmpliconEZ with total number of reads, top 90% mutation types and frameshift mutation rates in the representative animals injected with either *Shh*-guide RNAs #1, #2 or #3. The regions highlighted in red (Animal 1-*Shh*-guide RNAs #1), green (Animal 1-*Shh*-guide RNAs#2) and blue (Animal 1-*Shh*-guide RNAs#3) are the target sequences for each guide RNA. Each animal had numerous mutation types: deletions are depicted as hyphens and insertions are in lower case and red text. WT: wild type sequence, -Δ: nucleotide deletions and %: percentage reads for each mutation type represented against it.

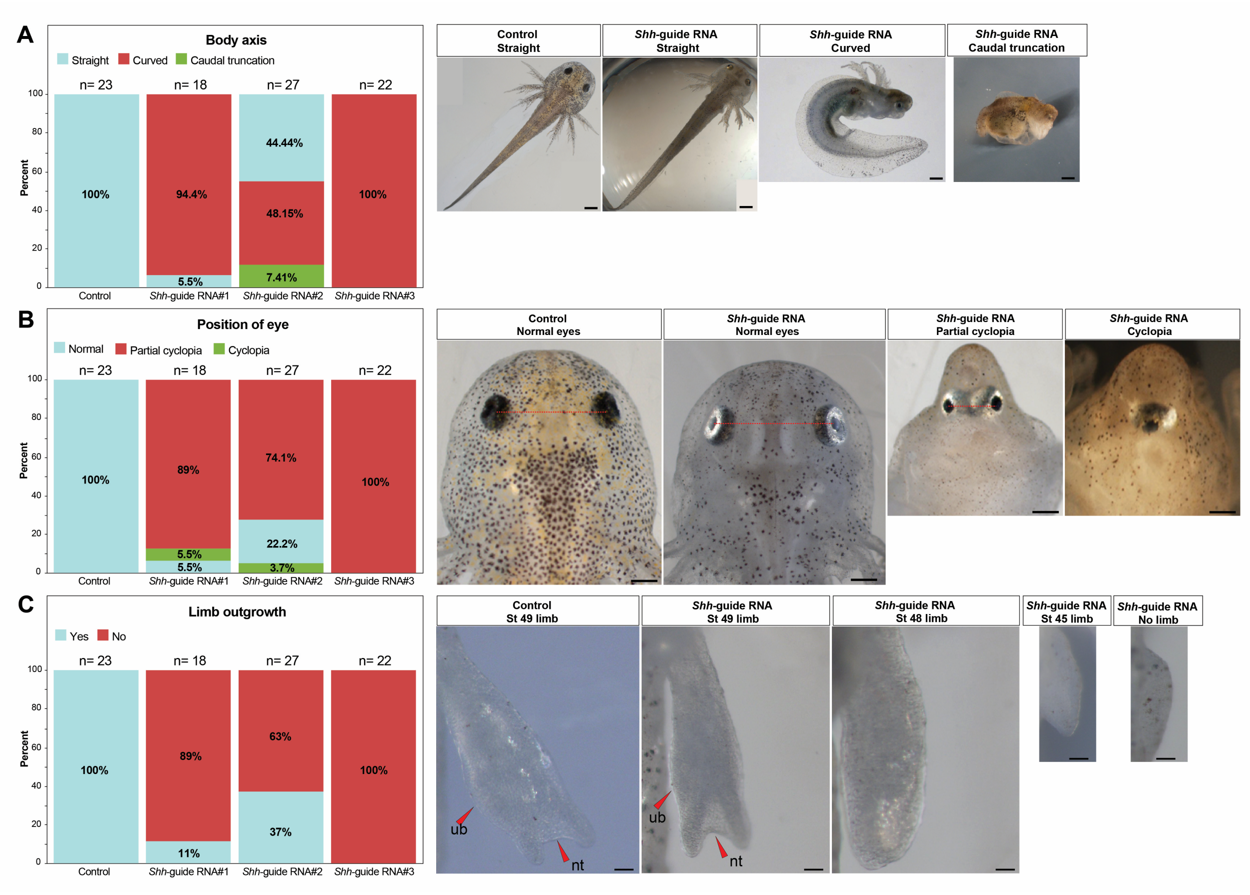

Figure S4. Three separate *Shh*-guide RNAs result in highly overlapping phenotypes in F0 crispants. **(A)** Various body axis phenotypes per guide RNA type in CRISPR control and *Shh* crispant larvae. **(B)** Various eye positions per guide RNA type in CRISPR control and *Shh* crispant larvae. Red dotted line: distance between the eyes. **(C)** Various limb phenotypes per guide RNA type in CRISPR control and *Shh* crispant larvae. n=23 for control larvae, n=18 for *Shh*-guide RNA#1 injected larva, n=27 for *Shh*-guide RNA#2 injected larvae and n=22 or *Shh*-guide RNA#3 injected larvae. Scale bar = 1 mm (for body axis), = 500 µm (for eye position) and =100 µm (limb stages). ub, ulnare bulge and nt, notch.

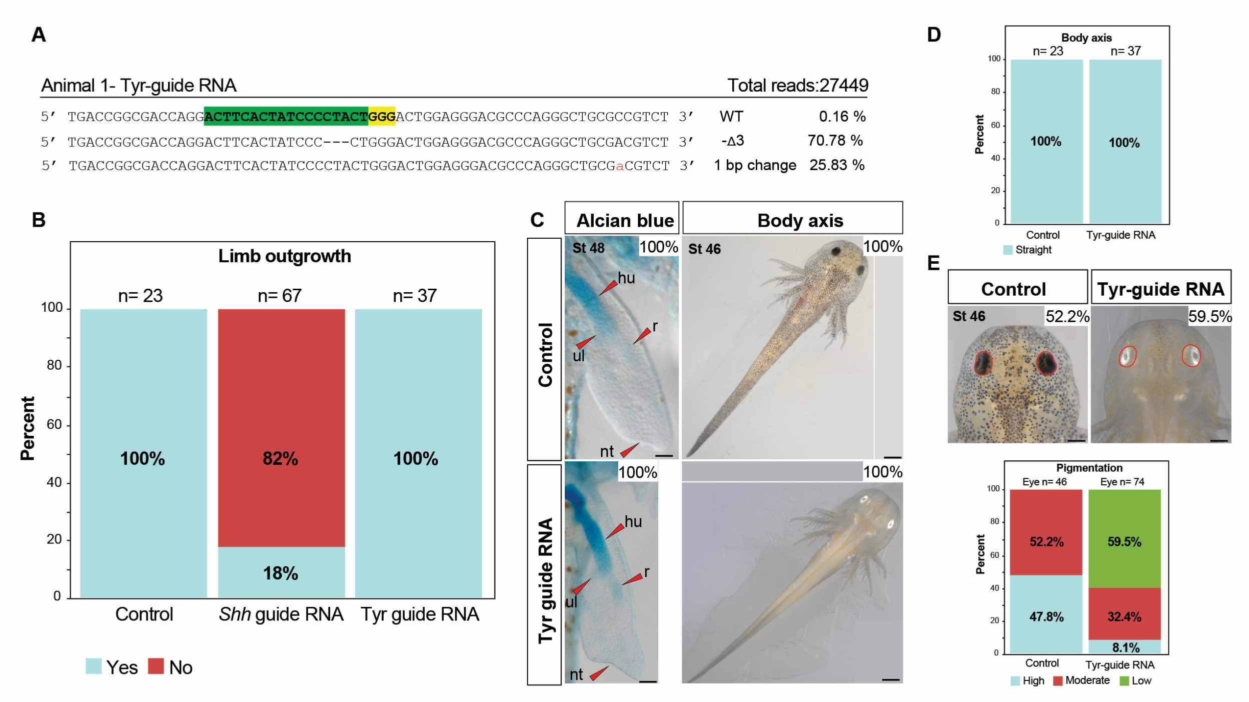

Figure S5. ***Tyrosinase* (*Tyr*) crispants do not show limb defects.** **(A)** Genotype of *Tyr* crispants analyzed using AmpliconEZ with total number of reads, top 90% mutation types and frameshift mutation rates in the representative animal. Deletions: hyphens and insertions: lower case and red text. WT stands for wild type sequence, -Δ represents nucleotide deletions and % reads for each mutation type is represented against it. **(B)** Percentage of CRISPR control, *Shh*- and *Tyr*-crispant larvae with limb bud outgrowth (n=23 for ethanol, =67 *Shh*-crispants and =37 *Tyr*-crispants). **(C, D)** Alcian blue staining (n=10 each) and body axis analysis (n=23 CRISPR control, =37 *Tyr*-crispants) for CRISPR control and *Tyr*-crispants. Scale bar = 1 mm (for body axis), = 500 µm (for Alcian blue staining). **(E)** Eye pigmentation (inside red dotted boarder) was calculated as pixel intensity using FIJI software and pixel intensity ranges were as follows: pixel intensity=221 to 148 (high), =147 to 74 (moderate) and =73-0 (low). n= 46 for CRISPR control and n=74 for *Tyr*-crispants. Scale bar = 500 µm. hu, humerus; ul, ulna; r, radius and nt, notch.

Supplementary tables

Table S1. List of primers used in this study

Experiment	Primers	Primer sequences 5' to 3'
WISH	Ptch1 Forward	GATCACAGAAGCTGGCTATGTAAAC
	Ptch1 Reverse	AAATATCAAGTCTGCAATCTTCGCG
	Fgf8 Forward	TTCACGTGCCTCACCTCCACTACCTCAGCA
	Fgf8 Reverse	ATATAAATTGTTCTTCTAAAAGTCCACTGG
	Gremlin1 Forward	TCGCCTGACACTACATAGC
	Gremlin1 Reverse	AGATGTGCAAAAGTTCAGAGAT
qRT-PCR	Fgf8 Forward	ATTAATTGTGGAAACGGACACCTTC
	Fgf8 Reverse	AATCAGCTTTCCCTTCTTGTTCATG
	Etv1 Forward	TCTTGGAAGAGTTCTTCTGAGTCAT
	Etv1 Reverse	CGTGTGAGAAATTGTAACGAGAGA
	Etv4 Forward	ACTATGCATACGATTCAGATGTTCC
	Etv4 Reverse	ATAGCCCTCCACGTTCATATACATT
	Gremlin1 Forward	GGACACCCAGAATACTGAGCA
	Gremlin1 Reverse	GTAGACCAATCGAAACATCCTGT
	Ptch1 Forward	TGTAGATCTGCTCCAATGCAAAC
	Ptch1 Reverse	CTGACCCGGAGTACTTGCAG
	Rlp32 Forward	AGGCTACTGGGAGTTTTAATAAGGA
	Rlp32 Reverse	AGATTACAGCACCCACTGTCTTTT
CRISPR/Cas9	DNA template for Shh guide 1	TTGAAATTAATACGACTCACTATAGGTCTACTGTGAGTACCCGGGTTTTAGAGCTAGAAATAGCAAGT
	DNA template for Shh guide 2	TTGAAATTAATACGACTCACTATAGGTACTCACAGTAGACCAGGGTTTTAGAGCTAGAAATAGCAAGT
	DNA template for Shh guide 3	TTGAAATTAATACGACTCACTATAGGTGACGCGTGTGTACCTGGGTTTTAGAGCTAGAAATAGCAAGT
	DNA template for Tyr guide	TTGAAATTAATACGACTCACTATAGGACTTCACTATCCCCTACTGTTTTAGAGCTAGAAATAGCAAGT
	Constant sequence	AAAAGCACCGACTCGGTGCCACTTTTTCAAGTTGATAACGGACTAGCCTTATTTTAACTTGCTATTTCTAGCTCTAAAAC
	gRNA-fw2 primer	TTGAAATTAATACGACTCACTATAGG
	DR274-rev	AAAAGCACCGACTCGGTGCCAC
	Shh genotyping primer forward	CGCGGCCCACCTCCTCTTTG
	Shh genotyping primer reverse	CGCCCAGCTGTGCTCCTCTATG
	Tyr genotyping primer forward	GCTCTTCCTGCTGCTCTGGGAG
	Tyr genotyping primer reverse	CTGCCAAGAAGCGAAGAAGGAG

Table S2. NGS results

**Animal Sample**	**Target locus**	**Target Reads**	**Mutant Reads**	**Mutant %**	**Genotype**	**Frameshift Mutant Reads**	**Frameshift Mutant %**
Control	*Shh*	73144	917	1.25	Homozygous WT	129	0.18
Shh-guide RNA#1	*Shh*	73801	73801	100	Homozygous Mutant	73739	99.92
Shh-guide RNA#2	*Shh*	38502	38467	99.91	Homozygous Mutant	37987	98.66
Shh-guide RNA#3	*Shh*	35642	35642	100	Homozygous Mutant	35382	99.27
Control	*Tyrosinase*	38044	57	0.15	Homozygous WT	53	0.14
Tyr-guide RNA#1	*Tyrosinase*	27449	19584	71.35	Heterozygous	34	0.12
